# Distinct gene expression program dynamics during erythropoiesis from human induced pluripotent stem cells compared with adult and cord blood progenitors

**DOI:** 10.1186/s12864-016-3134-z

**Published:** 2016-10-21

**Authors:** Alison T. Merryweather-Clarke, Alex J. Tipping, Abigail A. Lamikanra, Rui Fa, Basel Abu-Jamous, Hoi Pat Tsang, Lee Carpenter, Kathryn J. H. Robson, Asoke K. Nandi, David J. Roberts

**Affiliations:** 1 Radcliffe Department of Medicine, University of Oxford, Headington, Oxford, OX3 9DU UK; 2 National Health Service Blood and Transplant, John Radcliffe Hospital, Headington, Oxford, OX3 9BQ UK; 3 Department of Electronic and Computer Engineering, Brunel University London, Middlesex, UB8 3PH UK; 4 MRC Weatherall Institute of Molecular Medicine, University of Oxford, Headington, OX3 9DU Oxford, UK; 5 Distinguished Visiting Professor, The Key Laboratory of Embedded Systems and Service Computing, College of Electronic and Information Engineering, Tongji University, Shanghai, People’s Republic of China

**Keywords:** Erythropoiesis, Transcriptome, hiPSC, SMART and Bi-CoPaM

## Abstract

**Background:**

Human-induced pluripotent stem cells (hiPSCs) are a potentially invaluable resource for regenerative medicine, including the in vitro manufacture of blood products. HiPSC-derived red blood cells are an attractive therapeutic option in hematology, yet exhibit unexplained proliferation and enucleation defects that presently preclude such applications. We hypothesised that substantial differential regulation of gene expression during erythroid development accounts for these important differences between hiPSC-derived cells and those from adult or cord-blood progenitors. We thus cultured erythroblasts from each source for transcriptomic analysis to investigate differential gene expression underlying these functional defects.

**Results:**

Our high resolution transcriptional view of definitive erythropoiesis captures the regulation of genes relevant to cell-cycle control and confers statistical power to deploy novel bioinformatics methods. Whilst the dynamics of erythroid program elaboration from adult and cord blood progenitors were very similar, the emerging erythroid transcriptome in hiPSCs revealed radically different program elaboration compared to adult and cord blood cells. We explored the function of differentially expressed genes in hiPSC-specific clusters defined by our novel tunable clustering algorithms (SMART and Bi-CoPaM). HiPSCs show reduced expression of c-KIT and key erythroid transcription factors SOX6, MYB and BCL11A, strong HBZ-induction, and aberrant expression of genes involved in protein degradation, lysosomal clearance and cell-cycle regulation.

**Conclusions:**

Together, these data suggest that hiPSC-derived cells may be specified to a primitive erythroid fate, and implies that definitive specification may more accurately reflect adult development. We have therefore identified, for the first time, distinct gene expression dynamics during erythroblast differentiation from hiPSCs which may cause reduced proliferation and enucleation of hiPSC-derived erythroid cells. The data suggest several mechanistic defects which may partially explain the observed aberrant erythroid differentiation from hiPSCs.

**Electronic supplementary material:**

The online version of this article (doi:10.1186/s12864-016-3134-z) contains supplementary material, which is available to authorized users.

## Background

Definitive erythropoiesis in mammals replenishes the circulating pool of red blood cells (RBCs) and is controlled by intrinsic and extrinsic factors, notably cytokines that induce/select lineage commitment and differentiation from the hematopoietic stem cell (HSC). Two distinct programs of mammalian erythropoiesis have been elucidated through the use of human embryonic stem (ES) cells and murine studies to describe transcriptional and morphological changes during hematopoiesis [1–4]. In the first trimester, hematopoietic precursors in the fetal yolk sac follow a primitive erythropoietic program. In the second trimester, HSCs from the fetal liver and bone marrow yield enucleated erythrocytes via a definitive program of erythropoiesis.

Production of erythroid progenitors from hematopoietic stem/progenitor cells (HSPCs) in vitro recapitulates this definitive process using specific cytokines (IL-3, IL-6 and stem cell factor (SCF)), generating progenitors with megakaryocyte/erythroid potential (MEPs) and then committed erythroid cells. Initially, CD34^+^ cells barely proliferate as progenitors but are selected and/or programmed for expansion, becoming dependent on erythropoietin (EPO) and SCF for their survival [5].

The developing erythroid progenitors then expand rapidly as proerythroblasts undergo changes in morphology and cell surface phenotype, including acquisition of the lineage marker glycophorin A (*GYPA*, CD235a). After several rounds of expansion, maturation proceeds with vast synthesis of hemoglobin before chromatin condensation and enucleation produces reticulocytes that develop further in the bone marrow, circulation and spleen. Up-regulation of erythroid transcription factors (TFs) such as GATA1, SCL/TAL1 and KLF1, and down-regulation of TFs specific to other lineages, chromatin modifier proteins and enhancer elements, co-operatively form a transcriptional network that drives red cell development [6–8].

Erythroid cultures derived from human CD34^+^ progenitors in cord and/or peripheral blood have been used to describe physiological and pathological erythroid transcriptomes and associated TF activity [9, 10]. Erythroid cultures derived from embryonic stem cells or human induced pluripotent stem cells (hiPSCs) have also been investigated for their clinical potential [11–14]. These hiPSC lines have been used to model erythropoiesis in individuals with genetic disorders and suggest the possibility of deriving engineered transfusable RBC products or transplantable stem cells [15–22]. However, at the time of writing, no data has been generated to assess the fidelity of erythroid gene expression changes with time during erythropoiesis from hiPSCs in comparison with adult progenitors.

Red cells derived from hiPSCs can express many of the proteins required for normal erythrocyte function [23]. In vitro this includes hemoglobin A [14], and in vivo they are able to switch from fetal to adult hemoglobin [24]. However, expansion and enucleation rates from hiPSC-derived erythroblasts are much lower than observed with erythroblasts derived from adult or cord blood [12, 24]. The causes of these differences are unclear, but are presumably driven by differences in gene expression that can be identified in dynamic transcriptomic analyses.

Previously we have described the erythropoietic transcriptome in vitro using staged populations of sorted erythroblasts grown from adult peripheral blood progenitors [25]. This and other papers describing erythropoiesis from CD34^+^ progenitors [26–29] have typically yielded a relatively small number of staged samples for comparison. Transcriptome analysis of samples representing more stages through erythropoiesis (during a period of marked morphogenetic remodelling) improves the statistical power of bioinformatic approaches [28, 30, 31].

We therefore extended our prior observations by further subdividing erythropoietic cultures derived from adult hematopoietic progenitors to increase the number of staged populations studied that allowed use of our novel algorithms to cluster co-expressed genes in an unsupervised and tunable manner [30, 31]. These algorithms were used to define co-ordination of gene expression during erythropoiesis from adult, cord blood and hiPSC hematopoietic progenitors. With this approach we have been able to identify, for the first time, robust and specific patterns of gene regulation from hiPSC progenitors which differ markedly from those observed in adult- and cord-derived erythroblasts, and which may, at least in part, cause reduced proliferation and enucleation of erythroid cells from hiPSCs.

## Results

### Transcriptome analysis of adult erythropoiesis

CD34^+^ HSPCs from adult peripheral blood were cultured in SEM-F (Table 1 and Additional file 1: Figure S2A), which contained FBS, EPO, SCF, dexamethasone and IL-3. Triplicate samples representing 7 well-defined stages were obtained with distinct morphological and phenotypic characteristics (Additional file 1: Figure S2B). Each population corresponded to erythropoietic stages of differentiation sampled from day 0 to day 14 of culture. Cultures yielded ~10,000-fold expansion of the original CD34^+^ cells to pyknotic erythroblasts on day 14 (Additional file 1: Figure S2C).

**Table 1:** Standard Erythroid Media (SEM) components. Erythroid progenitors were cultured in two Standard Erythroid Medium modifications SEM-F or SEM-i as indicated in methods

Component added to IMDM (Biochrom)	Source	SEM-F			SEM-i			
		Phase I (Days 1-8)	Phase II (Days 8-11)	Phase III (Day 11 onward)	Phase I (Days 1-2)	Phase II (Days 2-8)	Phase III (Days 8-14)	Phase IV (Day 14 onward)
Fetal Bovine Serum (FBS)	Sigma	2 %	2 %	2 %	3 %	3 %	3 %	3 %
Penicillin	Sigma	100 U/ml	100 U/ml	100 U/ml	100 U/ml	100 U/ml	100 U/ml	100 U/ml
Streptomycin	Sigma	100 μg/ml	100 μg/ml	100 μg/ml	100 μg/ml	100 μg/ml	100 μg/ml	100 μg/ml
Glutamine	Sigma	4E-3M	4E-3M	4E-3M	4E-3M	4E-3M	4E-3M	4E-3M
Human AB serum	Sigma	3 %	3 %	3 %	2 %	2 %	2 %	2 %
Folic Acid	Sigma	10 μg/ml	10 μg/ml	10 μg/ml	10 μg/ml	10 μg/ml	10 μg/ml	10 μg/ml
Inositol	Sigma	40 μg/ml	40 μg/ml	40 μg/ml	40 μg/ml	40 μg/ml	40 μg/ml	40 μg/ml
Holotransferrin	Sigma	200 μg/ml	1 mg/ml	1 mg/ml	120 μg/ml	120 μg/ml	1 mg/ml	1 mg/ml
Human Recombinant Erythropoietin (Epo)	Janssen	3 U/ml	3 U/ml	3 U/ml	3 U/ml	3 U/ml	3 U/ml	3 U/ml
Insulin	Sigma	10 μg/ml	10 μg/ml	10 μg/ml	10 μg/ml	10 μg/ml	10 μg/ml	10 μg/ml
Monothioglycerol	Sigma	1.6E-4M	1.6E-4M	1.6E-4M	1.6E-4M	1.6E-4M	1.6E-4M	1.6E-4M
Cholesterol-Rich Lipids	Sigma	40 μg/ml	40 μg/ml	40 μg/ml	40μg/ml	40μg/ml	40μg/ml	40 μg/ml
Heparin	Sigma	3 U/ml	3 U/ml	3 U/ml	3 U/ml	3U/ml	3U/ml	3 U/ml
Human Stem Cell Factor (SCF)	R&D Systems	100 ng/ml	100 ng/ml	0	100 ng/ml	100 ng/ml	100 ng/ml	0
Dexamethasone	Sigma	1E-6M	0	0	1E-6M	1E-6M	1E-6M	1E-6M
IL3	R&D Systems	1 ng/ml	0	0	5ng/ml	5ng/ml	0	0
FLT3 Ligand	R&D Systems	0	0	0	20 ng/ml	20 ng/ml	20 ng/ml	0
IL6	R&D Systems	0	0	0	10 ng/ml	0	0	0
Thrombopoietin	R&D Systems	0	0	0	20 ng/ml	0	0	0

*IMDM* Iscove’s Modified Dulbecco’s Medium; *IL3* interleukin-3; *BSA* bovine serum albumin; *FLT3* Fms-like tyrosine kinase 3; *IL-6* interleukin-6

Data resulting from hybridisation of total RNA from these cells to Affymetrix HTA microarrays was analysed for differentially expressed genes as cells progressed through different erythropoietic stages (Additional file 1: Figure S2D).

**Fig. 1:**
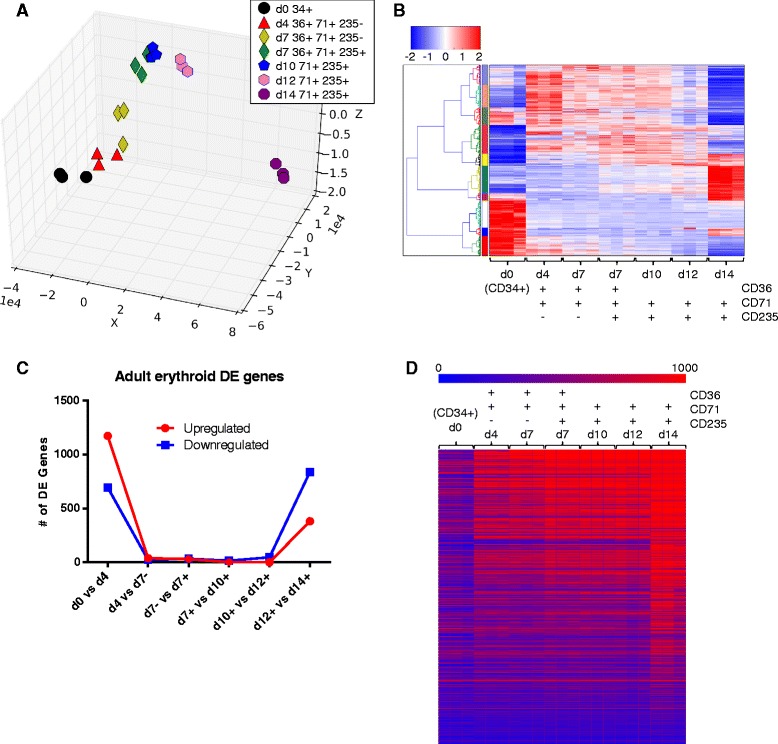
Gene expression during erythroid differentiation from adult stem cells in SEM-F. **a** PCA of differential gene expression in the triplicate AB FBS samples transforms the data into a series of uncorrelated variables made up from linear combinations and shows, in an unsupervised analysis, the progression of the differentiating erythroid cells through gene expression state-space. Genes reaching a minimum linear expression value of 100 in all replicates of at least one sample group were selected as differentially-expressed (DE) between any two stages during erythroid differentiation if they met the following criteria: *p* ≤ 0.01, fold change (FC) ≥ 2, B > 2.945 (Additional file 2: Table S1A). The union of all DE genes was used in the PCA. The Euclidean distances relating to this PCA are available in Additional file 19: Table S6. **b** Hierarchical clustering analysis of the differentially-expressed genes used in (A), clustering by gene only according to Euclidean distance. The colour bar on the left hand side denotes clusters of co-regulated genes. **c** Plot showing the number of differentially-expressed genes between consecutive populations in the adult FBS time-course data. **d** Genes described to be preferentially expressed in primary human erythroid cells [32] were examined in our current adult SEM-F dataset. Where these genes were expressed in our data, their expression pattern is shown as a hierarchical clustering, clustered by gene

Principal component analysis (PCA) demonstrated a large distance between the samples from day 0 and all later samples (Fig. 1a). Surprisingly, we detected relatively small distances between clusters of samples from progressive population types during the early phases of erythropoiesis (day 4, day 7^−^, day7^+^, and day 10). However, there is a more dynamic phase of gene expression changes late in maturation as cells prepare for enucleation (days 12 to 14) (Fig. 1a and Additional file 2: Table S1A, and S1B), consistent with our previous data [25]. Hierarchical clustering of the transcriptome data delineated well-defined patterns of gene expression changes that characterise erythropoiesis. This erythroid program is broadly segregated into 3 blocks of genes: one expressed at day 0 then repressed; another transiently up-regulated at days 4-10; and one other induced late in differentiation (Fig. 1b and Additional file 3: Figure S4). This pattern of transcriptional changes implied in the PCA and hierarchical clustering analysis was confirmed by enumeration of individual transcript expression changes through erythroid maturation (Fig. 1b and c and Additional file 3: Figure S4).

### Confirmation and comparison of transcriptomes from adult erythropoiesis

Our previously-described erythroid-induced genes [25] are predominantly up-regulated in our current data (Additional file 2: Table S1A, and Additional file 4: Table S2). Furthermore our dataset contains examples of upregulated erythroid specific genes, such as kruppel-like factor- erythroid (*KLF1*), which had not been identified in our previous dataset. Indeed, an additional 1573 differentially expressed genes are added whose regulation was not detected in our previous approach (Fig. 1b and Additional file 4: Table S2).

We also examined genes expressed preferentially in staged *ex vivo*-isolated primary human erythroid cells [32] for their expression in our data. These genes are indeed induced during elucidation of the erythroid gene expression program (Fig. 1d) and include important erythropoietic regulators such as *SCL*/*TAL1*, *NFE2*, *BCL2L1*, *BCL2L11*, *BCL6* and *SOX6*.

Gene ontology (GO) analysis identified early induction of genes encoding cell-cycle proteins including cyclins and cyclin-dependent kinases and the origin recognition/minichromosome licensing complex (Table 2). These data are consistent with the emergence of proliferative erythroblasts from the CD34^+^ HSPCs. Indeed, late in differentiation when proliferation declines, many of these transcripts encoding elements of the cell-cycle machinery are repressed while negative cell-cycle regulators such as *CDKN1B*/*p27KIP1* and *CDKN2C*/*p18* are induced (Additional file 2: Table S1A, and Additional file 4: Table S2). Thus taken together, these observations of staged populations suggest that we have captured the co-ordinated up- and down-regulation of overlapping gene expression programs relevant to cell-cycle control during erythropoiesis and as seen in primary erythroblasts *ex vivo*.

**Table 2:** Genes differentially expressed (DE) (B value at least 2.945, p-value below 0.01, fold change at least 2, and expression levels in all replicates at any population at least 100) during maturation of adult erythroblasts in SEM-F were selected as shown, and submitted to gene ontology analysis using GeneCoDis

Comparison	GO category	Corrected Hyp. *p* Value
Upregulated d0 to d4	Mitotic cell cycle	2e^−91^
Downregulated d0 to d4	Cytokine-mediated signalling	7e^−20^
	Antigen processing and presentation	7e^−18^
Upregulated d12 to d14	Nucleus/cell cycle arrest	7e^−6^
Downregulated d12 to d14	Purine metabolism	4e^−11^
	Ribosome biosynthesis	8e^−9^
	Mitotic cell cycle	7e^−8^

Significantly-scoring ontologies are shown, together with *p*-values presented as the more stringent corrected hypergeometric (Hyp) *p*-values produced by GeneCoDis

### Comparison of adult and cord blood erythroid programs

**Fig. 2:**
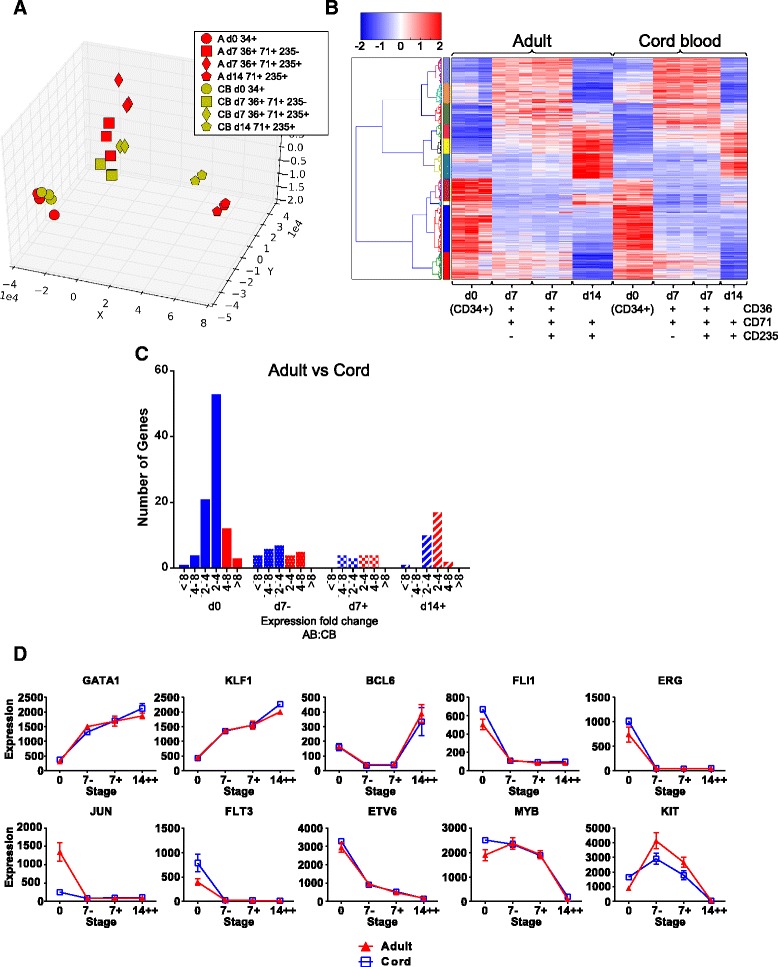
Gene expression during erythroid differentiation from adult and cord blood stem cells. **a** PCA of DE gene expression in cord blood- (CB-) derived differentiations in SEM-F. Genes were selected if they were DE between any two AB populations, or between any two CB populations (Additional file 5: Table S3A). The union of these two DE gene sets were then used to arrange the samples in the PCA, clustering the CB-erythroblasts together with the AB-erythroblasts shown in Fig. 1. CB-erythroblast populations were isolated with the same gating strategy used for AB-erythroblasts. The Euclidean distances relating to this PCA are available in Additional file 19: Table S7. **b** Hierarchical clustering analysis of the same AB or CB DE gene set, clustering by Euclidean distance and by gene. The colour bar on the left hand side denotes clusters of co-regulated genes. **c** The number of DE transcripts between AB-erythroblasts and the same CB-erythroblast population is shown to examine the fold change at each point during erythropoiesis. Blue bars depict genes that are expressed more abundantly in CB-erythroblasts, and red, in AB-erythroblasts. **d** Mean expression values of selected key erythroid genes during erythropoiesis in SEM-F, +/- standard error of the mean. ACTB and PAFAH1B2 were used to normalise the data since they were consistently expressed throughout erythropoiesis in this data (Additional file 4: Tables S2)

We wanted to establish whether similar patterns of gene expression could be observed during erythroid expansion from cord blood HSPCs cultured in the same media, SEM-F. We compared samples prepared at the most dynamic phase of gene expression between days 7 and 14 (Fig. 2 and Additional file 5: Table S3A and S3B).

First, developing erythroid cells from adult or cord HSPCs have broadly similar emergent transcriptomes: PCA shows adult peripheral blood and cord blood samples cluster together in domains separated by time from the day 0 CD34^+^ populations to the day 14 samples, but do not differ significantly by source (Fig. 2a). Hierarchical clustering analysis shows adult and cord blood samples largely co-regulate blocks of genes whose expression is similar in both cell types (Fig. 2b, Table 3 and Additional files 6 and 7: Figures S5A and B).

**Table 3:** Comparison of the gene expression changes between samples in the AB-erythroblasts and CB-erythroblasts during maturation

Expression change	Sample	d0 : d7-	d7- : d7+	d7+ : d14++
Upregulated	Adult	828 (60 % shared in CB)	30 (0 % shared in CB)	413 (34 % shared in CB)
Downregulated	Adult	710 (65 % shared in CB)	35 (0 % shared in CB)	1331 (37 % shared in CB)
Upregulated	CB	872 (53 % shared in Adult)	0 (0 % shared in CB)	174 (80 % shared in Adult)
Downregulated	CB	982 (56 % shared in Adult)	0 (0 % shared in Adult)	568 (86 % shared in Adult)

The number of DE genes from each source, and the degree to which these genes are similarly DE from the other source, is shown

**Fig. 3:**
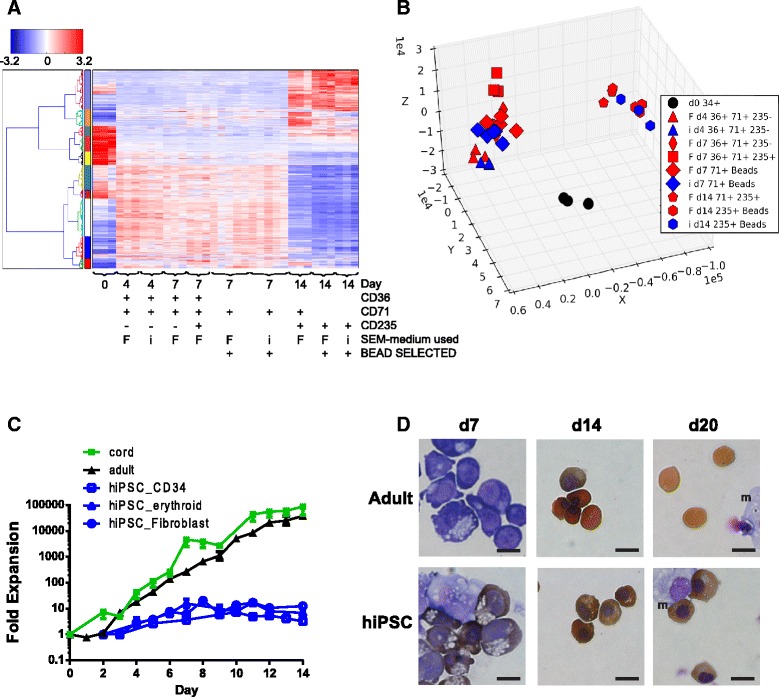
The gene expression profile of hiPSC derived erythroblasts is independent of the media used as evidenced by comparison of erythroid differentiation in SEM-i with SEM-F. **a** Hierarchical clustering analysis by Euclidean distance of adult erythroid differentiations performed using media for adult derived HSPCs (SEM-F), or hiPSC conditions (SEM-i). The gene set used contained genes which were DE during maturation in any of the two media settings (Additional file 20: Table S4). The colour bar on the left hand side denotes clusters of co-regulated genes. Samples cluster together by time and by the immunophenotype of the developing erythroid cells. There are no coherent subclusters formed according to the type of media used. **b** PCA of the samples shown in (A) with the same gene set. Populations are represented as follows: SEM-F, red symbols; SEM-i, blue symbols; d0 CD34^+^, black circles; day 4 CD36^+^CD71^+^CD235a^−^, triangles; day 7 CD36^+^CD71^+^CD235a^−^, thin diamond; day 7 CD36^+^CD71^+^CD235a^+^, square; day 7 CD71^+^ beads, fat diamond; day 14 CD71^+^CD235a^+^, pentagon; day 14 CD235a^+^ beads, hexagon. **c** Proliferation in SEM-i of erythroid AB-erythroblasts (black triangles), CB-erythroblasts (green squares) and hiPSC-erythroblasts (blue) where hiPSCs were specified from CD34^+^ peripheral blood (squares), erythroid cells (triangles) or fibroblasts (circles). Error bars indicate standard error of the mean of 3 or more cultures. **d** Morphological changes observed in erythroblasts of hiPSC and AB origin cultured in SEM-i. Representative images of Giemsa-benzidine stained cytospins of cultures on day 7, day 14 and day 20. Scale bar is ~10 μm. “m” is the stromal cell line MS-5. AB-derived differentiations, cultured further until day 20/21, were typically 70-80 % enucleated (see also Additional file 21: Figure S12), whereas the hiPSC-erythroblast cultures failed to enucleate

The difference in the number of genes differentially regulated between developing erythroid cells derived from adult and neonatal progenitors is greater at the CD34^+^ stage (day 0) than later (Table 3). This suggests convergence of similar fates in SEM-F despite apparent differences in the CD34+ compartment from adult and cord blood (Fig. 2c). We used GO analysis to identify groups of genes enriched for cellular components or molecular processes (Table 4) that were similar between cord and adult expression profiles. These findings are summarised in Additional file 5: Tables S3A and S3B. As well as key erythroid transcription factors *GATA1* and *KLF1* (Fig. 2d), the gamma globin gene, *HbG2* is also up-regulated equally in both profiles (Additional file 4: Table S2). Whilst non erythroid transcription factors and regulators are down-regulated in the first 7 days of differentiation, *FLI1*, *ERG*, *JUN*, *FLT3*, *ETV6*, notably *MYB* and *c-KIT* are down-regulated between days 7 and 14 (Fig. 2d and Additional files 6: Figure S5A and 7: Figure S5B). Once we had validated our in vitro culture system and shown the high similarity of adult and neonatal erythroid gene expression dynamics, we repeated the adult transcriptional analysis using SEM-i (Table 1), a medium that has been shown to yield maximal erythropoiesis from OP9 derived hiPSCs (see Methods). Crucially, adult erythroid development was largely unaffected by SEM-i when compared to SEM-F (Fig. 3a, b, Table 5; Additional file 8, Figure S6 and Additional file 9, Figure S7). Thus, we could reliably compare the elaboration of the erythroid program from adult and hiPSC-derived cells-of-origin in SEM-i.

**Table 4:** Gene ontology analysis of the DE genes in both AB-erythroblasts and CB-erythroblasts

Comparison	GO category	Corrected Hyp. *p* Value
Upregulated d0 to d7-	Cell cycle	3.9 e-^55^
	Porphyrin metabolism	1.3e-^9^
Downregulated d0 to d7-	Chemokine signalling	4.1e-^17^
	Antigen processing	1e-^11^
	Hematopoietic lineage	7e-^7^
Downregulated d7+ to d14	Ribosome biogenesis	1.6e-^8^
	Purine metabolism	2.7e-^8^
	Pyrimidine metabolism	5.5e-^5^

Highly-enriched ontologies are shown, together with the more stringent corrected hypergeometric (Hyp) *p*-values produced by GeneCoDis

**Table 5:** A breakdown of the number of DE genes during adult erythropoiesis in each medium type

Expression change	Medium	d0 : d7 BEADS	d7 BEADS: d14 BEADS	d0 : d4 FACS	d4 : d7- FACS	d7- : d7+ FACS	d7+ : d14++ FACS
Upregulated	SEM-F	1205 (89 % shared with SEM-i)	865 (70 % shared with SEM-i)	1174 (79 % shared with SEM-i)	38	30	416
Downregulated	SEM-F	953 (84 % shared with SEM-i)	1961 (81 % shared with SEM-i)	695 (82 % shared with SEM-i)	23	34	1335
Upregulated	SEM-i	1349 (70 % shared with SEM-F)	832 (73 % shared with SEM-F)	NA	NA	NA	NA
Downregulated	SEM-i	921 (86 % shared with SEM-F)	2324 (69 % shared with SEM-F)	NA	NA	NA	NA

*NA* not applicable

Numbers of genes up- or down-regulated between developmental stages in each medium setting are given, with the % similarly regulated in the other media

### hiPSC-derived erythropoiesis

The degree to which hiPSC-derived erythropoiesis reflects normal erythroid development is unclear. Certainly, erythroid cells derived from hiPSCs show reduced growth and enucleation compared to erythroid cells from adult or cord blood (Fig. 3c and d).

**Fig. 4:**
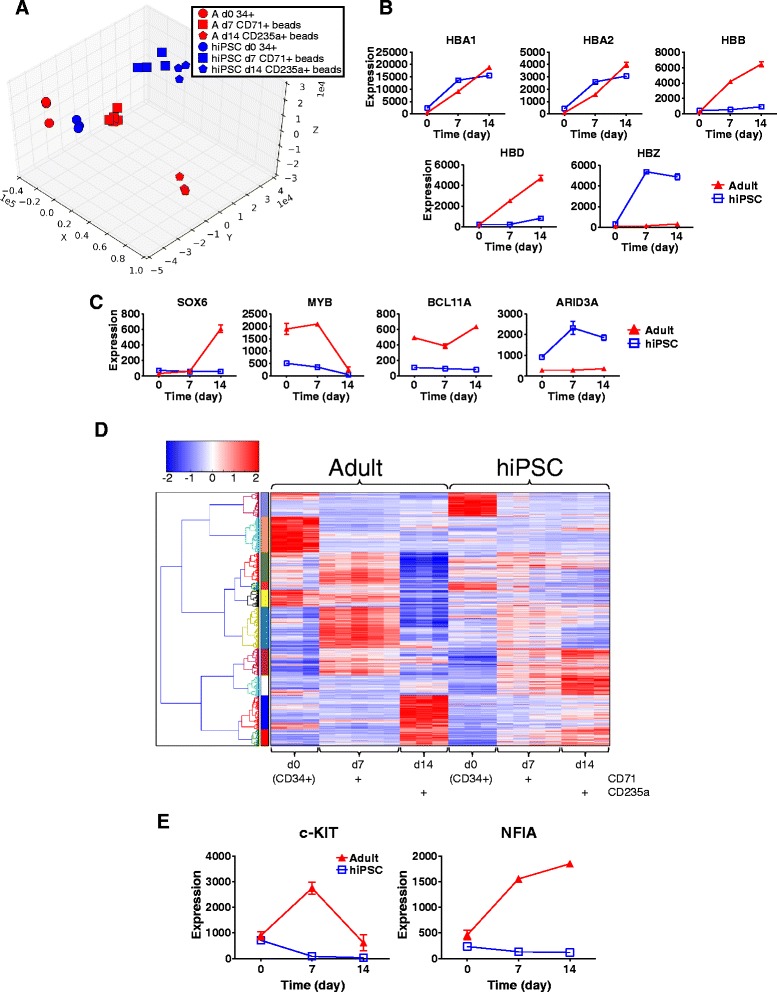
Elaboration of functional gene clusters in erythroid cells derived from adult and hiPSC progenitors in SEM-i. **a** PCA of genes differentially expressed in AB-erythroblasts (red symbols) or hiPSC-erythroblasts (blue symbols) during erythroid maturation in SEM-i. The gene set was any gene DE during maturation of erythroblasts from either source. The Euclidean distances relating to this PCA are available in Additional file 19: Table S8. **b** Expression profiles of globin genes in AB-erythroblasts and hiPSC-erythroblasts. Mean expression +/- standard error of the mean is plotted. **c** Expression profiles of genes encoding proteins with key roles in the regulation of erythroid development in the adult and hiPSC-derived settings. Mean expression +/- standard error of the mean is plotted. **d** Hierarchical clustering analysis by Euclidean distance of AB-erythroblasts and hiPSC-erythroblasts, clustered by gene only. The colour bar on the left hand side denotes clusters of co-regulated genes. **e** Gene expression profiles for c-KIT and NFIA in AB-erythroblasts and hiPSC-erythroblasts. Mean expression +/- standard error of the mean is plotted. Given these findings, we validated the microarray-based gene expression measurements of selected transcripts using quantitative RT-PCR (in Additional file 13: Figure S10B)

For comparison with the AB- and CB-erythroblast data, we examined transcriptional changes during erythropoiesis from three different hiPSC lines in SEM-i (Additional file 10: Table S5A and S5B). PCA analysis revealed that the hiPSC-derived samples clustered quite separately from the adult samples (Fig. 4a).

Erythroblasts derived from hiPSCs up-regulate genes encoding proteins involved in heme biosynthesis including *CPOX*, *PPOX*, *FECH*, *HMBS*, *UROD*, *ALAS2*, and *SLC25A39* somewhat earlier than the adult-derived cells, where expression of these genes peaked later, between days 7 and 14 (Additional file 11: Figure S8). Concerted up-regulation of cell-cycle genes in the adult samples was noted by day 7, but expression of this set of genes was more limited in intensity and breadth in the hiPSCs (Additional file 12: Figure S9).

Although we observed similar expression of the gamma globin gene HBG2 in erythroblasts from adult and hiPSC progenitors (Additional file 4: Table S2), a significant clue to the different erythropoietic program observed from hiPSCs was the apparent development of a primitive erythropoietic globin profile, as described previously in hiPSCs [18], and in human embryonic stem cells [33]. Whereas adult-derived cells gradually up-regulated *HBB* and *HBA*, hiPSC-derived cells expressed *HBA* and the embryonic globin *HBZ* (Fig. 4b). In contrast the adult-derived erythroblasts neither significantly expressed nor appreciably induced *HBZ*, consistent with a definitive erythropoietic profile (Additional file 13: Figure S10B).

Together, these data strongly suggest that these hiPSC-derived erythroblasts follow the embryonic or primitive program of hematopoiesis which is significantly different from the gene expression program delineated during erythroid development from adult HSPCs.

### Functional gene clusters

We and others [12, 18, 23] have noted that hiPSC-derived erythroblasts fail to enucleate as efficiently in vitro as adult-derived cells (Fig. 3d). Subsequently, we set out to identify clusters of genes whose expression correlated with these functional defects. First, we closely examined the expression of genes that regulate and co-ordinate erythroid development or have key functional roles in erythroid cells across all three datasets. We noted that transcripts such as *GATA1*, *TAL1*, *KLF1*, *EPOR* and *ANK1* are induced in both hiPSC-derived erythroblasts and in the adult setting (Additional file 4: Table S2 and Additional file 14: Figure S10A); other regulatory genes such as the AP-1 components *JUN*, *JUNB*, *JUND*, *FOS* and *FOSB* are expressed more highly in the adult cells prior to erythroid differentiation – thereafter expression dynamics are similar in adult and hiPSC-derived erythropoiesis (Additional file 14: Figure S10A).

In keeping with their apparent primitive erythropoietic globin gene expression profile, the key erythroid TFs *SOX6*, *MYB* and *BCL11A* are all expressed at much lower levels in hiPSC-derived erythroblasts compared with adult blood (Fig. 4c). We validated expression of these genes and others by quantitative RT-PCR (Additional file 13: Figure S10B). In murine erythropoiesis, these TFs are expressed in definitive blood cells (reviewed by Palis [34]) and function in switching globin expression from fetal to adult hemoglobin [35–37]. Finally, *ARID3A* expression, specific to primitive erythropoiesis [38], is poor in adult- but strong in hiPSC-derived erythropoiesis (Fig. 4c and Additional file 13: Figure S10B). Gene expression program differences visible in the PCA (Fig. 4a) are also evident in Hierarchical clustering analysis (Fig. 4d).

**Fig. 5:**
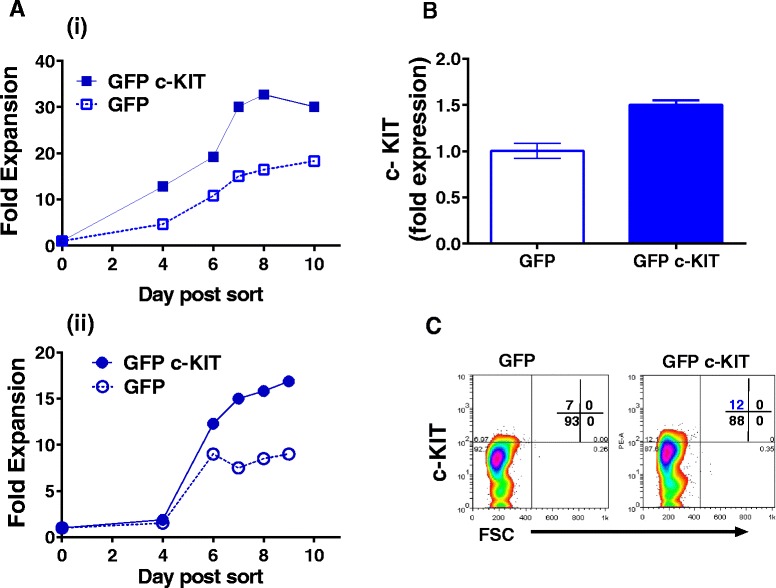
Transduction of hiPSC hematopoietic progenitors with lentivirus expressing c-KIT enhances erythroblast expansion. **a** Proliferation of hiPSC-erythroblasts transduced with either the GFP tagged lentiviral construct (GFP c-KIT) or with the control vector (GFP) was monitored after selection of GFP positive cells by FACS on day 2 of hiPSC erythroblast culture. Results from 2 independent experiments are shown in (i) and (ii). **b** Increased expression of c-KIT in erythroblasts grown from hiPSC progenitors transduced with the GFP c-KIT lentivral construct. Error bars are SDs of quadruplicate measurements by semi-quantitative PCR from cells taken on day 10 of culture showing a 1.5 fold increase that is comparable to the increase in cell expansion. **c** Protein expression of c-KIT at the surface of erythroblasts grown from hIPSC progenitors transduced with the GFP-c-KIT or control vector expressing GFP alone. GFP-c-KIT erythroblasts collected at the end of experiment (i) show a small increase in c-KIT expression where mean fluorescence intensity (MFI) is 47 compared with control MFI of 37 and the proportion of c-KIT positive cells is also modestly increased (highlighted in blue)

We noted in relation to the radically different proliferation of adult and hiPSC-derived erythroblasts (Fig. 3c) that the key erythroid genes *KIT* and *NFIA* [39] are expressed less in hiPSC-derived erythroid cells than in the adult-derived setting (Fig. 4e and Additional file 13, Figure S10B). We postulated that low expression of c-KIT in hiPSCs versus adult erythroblasts might be responsible for the failure of the hiPSCs to proliferate in response to SCF in the culture media. Therefore we utilised a lentiviral expression construct to enforce c-KIT expression in hiPSC-derived progenitors. Transduced cells were then isolated by flow sorting and their proliferation tracked. We found that cells transduced with c-KIT proliferated more than cells transduced with the control vector (Fig. 5a) which offers mechanistic support for potentially improving erythroblast yield via signalling through c- KIT.

### Upstream TFBS analysis from gene clusters and erythroid regulatory genes

**Fig. 6:**
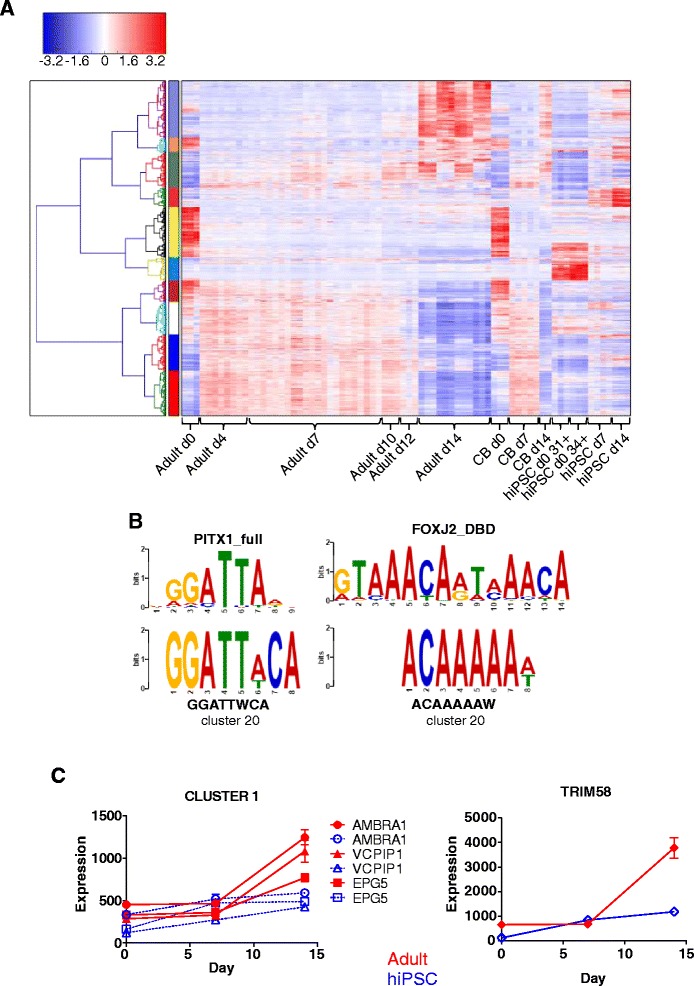
Differential expression of co-ordinately regulated genes relevant to erythroid expansion and terminal differentiation identified using SMART. **a** Standard hierarchical clustering analysis by Euclidean distance of the union of DE genes from all cells of origin, in all media, clustered by gene only, of all samples utilised in the study, regardless of medium type or cell-of-origin, to visualise clusters of robustly co-regulated genes. The colour bar on the left hand side denotes clusters of co-regulated genes. **b** Transcription factor binding site analysis of 1kb of genomic DNA sequence upstream of the TSS for genes in the DNA repair/cell-cycle cluster. Matches from MEME/TOMTOM analysis are depicted: upper panels are motifs from the database; lower panels are the enriched motif detected within cluster 20 from our SMART analysis. Vertical axes are scaled to 2 bits in all images, and horizontal axes show sequential bases in the relevant motifs. **c** Examples of genes in SMART cluster 1, important in autophagic processes, which are differentially expressed between AB-erythroblasts and hiPSC-erythroblasts during the last phases of differentiation

Given the biological differences between the terminal erythroid cells produced from different HSPCs, we proceeded to utilise SMART (splitting merging awareness tactics) [31] to allow us to identify co-ordinately-regulated clusters of genes which might be relevant to the differences in enucleation and proliferation observed in hiPSC differentiation compared to adult or neonatal stem/progenitor differentiation (Fig. 6a).

We examined clusters containing more than 30 genes for evidence of differential regulation between adult and cord blood samples and the hiPSC-derived cells. Several clusters were identified that fit this profile (Fig. 6a and Additional file 6: Figure S5A and Additional file 15: Figure S11).

These differentially-regulated clusters (10, 13, 16, 17, 19–24, 26–29, 32–35, Additional file 15: Figure S11) were scrutinised for genes with potential functional roles in either proliferation or enucleation. Examining the functional clusters for enriched gene ontologies was largely unproductive, partially due to the small number of genes contained within each cluster. However, we observed the highly significant co-regulation of genes involved in DNA repair processes in cluster 20, together with other genes involved in cell-cycle regulation. These genes (*FANCA*, *FANCB*, *FANCC*, *FANCD2*, *BRCA2* and *RAD51*) were examined for conserved upstream transcription factor binding motifs, and were found to be associated with enrichment of putative PITX and FOXJ motifs (Fig. 6b), suggesting putative regulators of expression for this functional gene cluster. Moreover, PITX1 expression increased steadily during erythropoiesis from adult and cord blood-derived cells, but failed to increase during hiPSC-derived erythropoiesis (Additional file 4: Table S2) confirming the absence of a known cell cycle regulator in hiPSC.

Finally, the marked vacuolisation seen in hiPSC-erythroblasts (Fig. 3d) was investigated, as this could indicate defects in the autophagolysosomal pathway that are critical to terminal differentiation of erythroblasts to reticulocytes [40, 41]. We found that many of the genes that encode for proteins that regulate autophagy were in cluster 1 with significantly reduced expression in hiPSC-erythroblasts on day 14 when compared with adult-erythroblasts (Fig. 6c). For example, one of these genes (VCPIP1/p97) is required for the segregation of organelles into lysosomal compartments, removal of autophagolysosomes enriched with light chain 3 [42] and regulation of mitosis [43]. Another (TRIM58) facilitates erythroblast enucleation by inducing dynein degradation [44]. These profiles strongly suggest dysregulation of the lysosomal degradation pathway in erythroblasts derived from hiPSCs.

## Discussion

The work described substantially extends our prior description of human erythropoiesis [25], offering a more fine-grained analysis and comparison of the gene expression program from three erythroid progenitor sources. Interestingly, 32 % of the newly identified differentially expressed genes are captured in SMART clusters. These include 168 genes co-ordinately regulated with one of the erythroid regulators GATA1, TAL1, KLF1 and EPOR. An example is co-clustering of *GSK3A*, *MTF1*, *ZNF175*, *MRFAP1L1*, *REXO2* and *LIN37* with KLF1 in cluster 25 (Additional file 4: Table S2 and Additional file 15: Figure S11) therefore allowing for the potential to identify novel regulators during erythropoiesis in vitro.

Gene expression analysis of adult or cord data revealed very similar gene expression programs, irrespective of culture media or cell origin, consistent with a previous report of modest differences between fetal and adult erythroid gene expression programs [45, 46]. However, comparison of gene expression during erythroid differentiation from hiPSCs and adult blood showed substantially different modules of co-regulated genes that correlate with phenotypic differences between the generated erythroid cells (Figs. 4, 5, ﻿and 6﻿; Additional file 16).

We selected our final samples based on their proximity to the point of nuclear extrusion and enucleation, after which comparative studies would require post-transcriptional or proteomic analyses [23, 47]. Thus we are able to identify gene expression dynamics relevant to the condensation, movement and eventual extrusion of the nucleus from the developing erythroid cell, a poorly understood but critical process for translational applications of in vitro erythropoiesis [48].

The contribution of EPO to gene expression is confirmed by early up-regulation of *TIMP1*, regulated by EPO via the classical erythroid TF GATA1 [49]. We capture the emergence of the erythroid program, with up-regulation of erythroid-associated genes such as the TFs *GATA1*, *TAL1*, *KLF1*, *BCL2L11* and *GFI-1B* (Additional file 4: Table S2) and down-regulation of the TFs *FOS*, *FOSB*, *JUN*, *JUNB* and *JUND* (Additional file 4: Table S2 and Additional file 14: Figure S10A) which may down-regulate the AP-1 transcription factor complex. We also identified the induction of known direct transcriptional targets of GATA1 and KLF1 such as *ALAS2* (Additional file 6: Figure 5A) [50]. So our data comparing day 0 and day 4 samples tracks the emergence of the erythroid program under control of known key regulators. In contrast, there are fewer differentially expressed genes between day 4, 7, 10 and day 12 samples, thus indicating a stable pattern of gene expression as early erythroblasts undergo rapid cell division.

Crucially, our data from hiPSCs showed that expression of the key regulators *SOX6*, *MYB* and *BCL11A* and the globin genes are quite consistent with specification of primitive erythropoietic cells [36, 37, 51]. Such observations of a putative primitive erythroid gene expression program in the hiPSC setting, although known from globin profiling [6], have not until now been described dynamically during erythropoiesis. The program-level of expression changes we describe here may have implications for generation of clinically-useful in vitro-derived RBC products. Whilst deficiency in expression of *BCL11A*, and expression of fetal globin, is therapeutically attractive for treatment of hemoglobinopathies (reviewed in [52]), primitive erythroid cells do not efficiently enucleate in vitro without the support of macrophages [4, 53]. In vivo, enucleating primitive erythroid cells are in close contact with macrophages in the fetal liver or human placenta or may enucleate in the circulation [3, 4]. Hence, it is not surprising that suspension-culture erythroid cells derived from hiPSCs fail to efficiently enucleate in vitro in our experiments. Stimulating enucleation may become possible, but we saw no enucleation when our hiPSC derived erythroid cells were co-cultured with a murine stromal cell line known to support enucleation [54] (Fig. 3d).

One report has suggested that the enucleation deficiency in these hiPSC lines may be due to the absence of cytoskeletal proteins such as α-catenin and β-tubulin, that subsequently disrupt remodelling of the cell membrane during enucleation [23]. Remarkably, some enucleation is observed from hiPSC-derived hematopoietic cells specified by certain TF combinations [55]. These authors reported definitive erythropoiesis in vivo following transplantation of hiPSC lines specified via a combination of constructs coding for *ERG*, *HOXA9*, *RORA*, *SOX4* and *MYB*. Interestingly, reported globin switching of nucleated hiPSC-derived erythroid cells following transplantation in vivo implies that transition to a definitive globin gene expression program is dependent on contextual cues [24, 55]. This has been supported by in vitro induction of hemoglobin A using specific stromal layers [14].

Derivation of a definitive erythroid program from hiPSCs in vitro potentially presents a significant challenge as key regulators of this switch from the primitive program have not been identified. More fruitful approaches arise from direct specification of the cells emerging from embryoid bodies to the definitive lineage [24, 56]. An alternate route to successful specification of hiPSCs to the definitive lineage involves inhibition of activin/nodal signaling and activation of the WNT/β-catenin pathway [57].

Within our data, there is some evidence that the WNT/β-catenin pathway may contribute to the aberrant phenotype observed when erythroblasts are derived from hiPSC. Among the genes that encode key players within this pathway, i.e., the Frizzled receptor family (*FZD4, 7* and *8*), *LR6*, *AXIN* and *APC*, only *GSK3A* is differentially regulated between adult and hIPSC derived erythroblasts. GSK3α (glycogen synthase kinase 3 alpha) forms a complex with proteins encoded by *AXIN* and *APC* to induce ubiquitination of β-catenin and inhibit continued stem cell expansion (reviewed in [58, 59]. This protein also phosphorylates and inhibits the TFs MITF, TFEB and TFE3 that upregulate genes required for the formation of lysosomes (see [60] for review). Intriguingly, in our dataset *GSK3A* is significantly upregulated in adult derived erythroblasts compared with those from hiPSC throughout culture (Additional file 10: Table S5B) and its expression co-clusters with erythroid regulators (Additional file 4: Table S2 and Additional file 15: Figure S11). Therefore, determining whether the post translational control of TFs by GSK3*a* in erythroblasts regulates expression of the autophagolysosomal genes found in cluster 1 of our SMART analysis (Fig. 6) may shed more light on how to overcome enucleation deficiencies in erythroid cultures from hiPSC.

It is possible that re-specifying emerging hematopoietic cells into a definitive fate correlates with an increased rate of spontaneous enucleation for in vitro erythropoiesis without helper cells. However apparently definitive erythroid cells generated from hiPSC (expressing fetal hemoglobin) still show enucleation that is 30 % of that observed in erythroid cultures derived from cord blood HSPCs [56]. Therefore, further analysis of the association between persistent vacuolisation and aberrant expression of genes in hiPSCs that are involved in protein degradation, lysosomal clearance and cell-cycle checkpoints is required to reveal how to enhance reticulocyte production in vitro.

Erythropoiesis from hiPSCs is always limited by the relative lack of proliferation compared to that seen in adult and neonatal erythroblasts. We noted that *c-KIT* was not induced during hiPSC-derived erythropoiesis, but was in adult and cord blood-derived cells where *c-KIT* expression resembled that in primitive erythroblasts [61]. Failure to respond to SCF might be predicted to confer a profound difference in proliferative response from hiPSC-derived erythroblasts, as response to SCF present in the medium during the first 11 days of culture would stimulate adult and cord blood erythroblast proliferation in SEM-i (Fig. 3). Notably, reduced expression of *c-KIT* resulting from mir221 and mir222 action is associated with markedly reduced erythroid cell proliferation [62]. Enforced expression of *c-KIT* in hiPSCs did appear to allow moderately increased proliferation, consistent with a role for c-KIT signalling in the much greater proliferative responses of adult cells to SCF in the culture medium. Understanding how to more efficiently express functional cell surface c- KIT in hiPSCs will be key to further investigating this important potential axis for intervention during in vitro differentiation protocols.

Other work has shown that c-KIT stabilises β-catenin expression [63] which forms a complex with LEF-1 to induce cyclin D1 expression [64]. Both cyclin D1 and LEF-1 are significantly downregulated in hiPSC erythroblasts (Additional file 10: Table S5B) suggesting that other regulators of cyclin D1 or LEF-1 should also be studied to further improve proliferation of hIPSC derived erythroblasts.

We note that erythroblasts derived from apparent definitively-specified hiPSCs [56] still demonstrate a profound proliferation defect; it is unclear whether *c-KIT* or *GSK3-a* expression were induced during erythropoiesis in this setting, further demonstrating the value of our dynamic gene expression analysis compared to steady-state observations.

## Conclusions

The comparative transcriptomics of erythroid cells derived from hiPSC, neonatal and adult progenitor cells have been highly informative but only partially represent the orchestration of protein expression required to produce viable and functional mature erythroid cells. Tissue-specific splicing of pre-messenger RNA (mRNA) produces isoforms of cytoskeletal and transport proteins that are required for function of mature erythrocytes [65, 66]. Alternative splicing can also introduce premature termination codons and such splicing switches appear to exert dramatic changes during the latter stages of RBC development [67, 68]. Moreover, production of microRNAs that themselves affect transcript levels are also known to differ between these developmental programs. These post-transcriptional levels of control are being analysed separately (manuscripts in preparation).

The data presented in this paper and detailed in the Supplementary Files highlight the disparities between erythropoiesis in vitro from diverse stem/progenitor cell origins and have generated a series of hypotheses and resources for further dissection of the programs of erythroid development.

## Methods

All reagents were obtained from Sigma Aldrich unless stated otherwise. Peripheral blood mononuclear cells were obtained from blood donated to the National Health Service Blood and Transplant (NHSBT; http://www.nhsbt.nhs.uk/) following written consent and their use approved by the National Health Service Oxfordshire Regional Ethical Committee.

### Cell culture

Human primary differentiating erythroblasts derived from adult peripheral blood lymphocyte cones (AB) (NHS Blood and Transplant) or cord blood (CB) were obtained with informed consent in accordance with the Declaration of Helsinki.

Erythroblasts were cultured using a three-phase liquid culture system [69] with modifications (Table 1).

Erythroid progenitors were cultured in two Standard Erythroid Medium modifications: SEM-F (used for AB-erythroblasts and CB-erythroblasts), or SEM-i (used for AB-erythroblasts and hiPSC-erythroblasts). Briefly, mononuclear cells were obtained from AB or CB by Ficoll-Hypaque density-gradient centrifugation. CD34+ erythroid progenitors were isolated using magnetic beads (Miltenyi) and their purity (>90 %) verified by flow cytometry. Cells were seeded at 2 x 10^5^ cells/ml in standard erythroblast culture medium containing 2 % Fetal Bovine Serum (FBS) (SEM-F). Cells were washed to remove cytokines between media changes. HiPSCs were established from fibroblasts or hematopoietic cells transduced with *OCT4*, *SOX2* and *KLF4* [14]. Erythroblasts were generated by modifying a published protocol [18] in medium optimised for hiPSC-derived erythroblast culture (SEM-i) (Table 1). Briefly, the co-cultured hiPSCs and OP9 cells were harvested after 8 days and disaggregated as previously described [18]. Cells were typically cultured for 14 days in the four-phase SEM-i culture protocol. Before transfer from Phase I SEM-i to Phase II, hiPSC-derived erythroblasts were gently disaggregated by pipette propulsion before separation of hematopoietic cells from OP9 cells using 20% Percoll gradients (Additional file 17: Figure S1). Some cultures were extended to day 21 to assess enucleation. HiPSC-derived erythroblast cultures were co-cultured with the murine stromal cell line, MS5, in SEM-i Phase IV medium (Table 1). Extended culture of erythroblasts derived from AB continued in Phase III SEM-F. Cell density did not exceed 10^6^ cells/ml throughout culture.

### Isolation of enriched populations

Cells were separated into enriched populations based on cell surface expression of CD34, CD36, CD71 and CD235a using flow cytometry or magnetic beads (Additional files 1 and 18). AB- and CB-derived erythroblasts were stained and sorted on a MoFlo II (Beckman Coulter) using anti–CD36-PE (BD Biosciences; for erythroblasts cultured for between four and seven days), anti–CD71-FITC (Dako), anti–CD235a-APC (BD Biosciences; for erythroblasts cultured between four and 12 days), and anti–CD235a-RPE (Dako; for erythroblasts cultured for 14 days). Cells were cultured from three independent samples for each population isolated. Triplicate samples of total CD34+ cells were also obtained on day 0 for RNA extraction.

Fluorescence-activated cell sorting (FACS) was used to isolate triplicate samples of discrete populations of cells similar in maturity and lineage. Debris and dead cells were excluded by examining forward scatter and DAPI-staining. A pulse-width gate in side scatter was applied to exclude doublets. Sort gates were defined by first setting a gate on the expression profile of the population of interest: CD36 + CD71+ on day 4, CD36 + CD71 + CD235a- and CD36 + CD71 + CD235a + on day 7 (day 7- and day 7+), and CD71 + CD235a + on days 10, 12 and 14 (day 10+, day 12+ and day 14+). Representative plots are shown in Additional file 1: Figure S2. These gates were then applied to the forward scatter versus side scatter dot plot to define a population of cells showing similar size. At day 0, AB-, CB- and hiPSC-derived HSPCs were isolated using CD34-beads (Miltenyi). HiPSC-derived hematopoietic progenitors were also isolated using CD31 beads at day 0 to incorporate all possible sources of erythroid progenitors. As insufficient cell numbers were obtained for FACS sorting of erythroblasts derived from hiPSCs, CD71 and CD235a-specific magnetic beads were used to isolate hiPSC-erythroblast populations at days 7 and 14 respectively. AB-erythroblasts were isolated using these beads for direct comparison with hiPSC-derived erythroblasts. The purity of each FACsorted or bead sorted population was typically > 90 %. The sort gates for isolated populations are shown in Additional files 1 and 18.

Maturation of erythroblasts was monitored by staining cytospin preparations from 5x10^4^ cells with Wright-Giemsa and assessed using light microscopy. To visualise hemoglobin expression, cytospins were stained with 1 % O-dianisidine in methanol and counterstained with 10 % Giemsa (BDH) (Additional file 1: Figure S2B).

### RNA extraction

Total RNA including miRNA was extracted using mirVana (Thermofisher) according to the manufacturer’s instructions. RNA quality and quantity was assessed using a Qubit fluorimeter (Thermofisher Scientific), a NanoDrop spectrophotometer (Thermo) and an Agilent Bioanalyzer 2100 (Agilent Technologies). RNA integrity numbers were in the range 8.7-10. Before target preparation for hybridisation to Affymetrix Human Transcriptome 2.0 (HTA2.0) arrays (Affymetrix), RNA was treated with Turbo DNA-Free DNAse (Thermofisher Scientific).

### Array hybridisation

The target was prepared from 100ng total RNA for hybridisation to Affymetrix GeneChip Human Transcriptome 2.0 ST microarrays (HTA2) using the Ambion WT protocol (Thermofisher Scientific) and Affymetrix labelling and hybridisation kits (Affymetrix). Labelled DNA mean yield was 13.5 μg (minimum: 8.5 μg; maximum: 20.5 μg). HTA2 arrays were hybridised with 5 μg of labelled DNA.

The Affymetrix GeneChip Fluidics Station 450 was used to wash and stain the arrays with streptavidin–phycoerythrin, according to the standard protocol for eukaryotic targets (IHC kit, Affymetrix). Arrays were scanned with an Affymetrix GeneChip scanner 3000 at 570 nm.

### Quantitative PCR

Validation of array data to confirm expression of genes was achieved following DNAse treatment of RNA and synthesis of cDNA using either the High Capacity cDNA Reverse Transcription Kit or the SuperScript® VILO™ cDNA Synthesis Kit (both from ThermoFisher Scientific). Semi-quantitative polymerase chain reaction (qPCR) was performed using TaqMan reagents and assays (ThermoFisher Scientific). Data was normalised using ACTB and PAFAH1B2 as reference genes since they were consistently expressed throughout erythropoiesis in our data (Additional file 4: Table S2). This qPCR data is shown in (Additional file 7: Figure S5B) and (Additional file 13 :Figure S10B).

### Enforced KIT expression in hiPSCs

Emerging hiPSCs at the point of disaggregation from OP9 co-culture were transduced with GFP+ lentivirus expressing the c-KIT open reading frame, or the empty vector (LV-165; both Genecopoiea) packaged essentially as described elsewhere [70]. HiPSCs were cultured as described above for three days before sorting of GFP+ CD235+ erythroid cells using FACS. The sorted hiPSCs were cultured on as described in 50 μl of medium in 96-well format and counted at intervals of 1-2 days. Cultures were fed 1:1 with fresh medium if they exceeded 3 x 10^5^ cells/ml. At the end of culture, cells were collected to measure c-KIT (PE conjugated anti-CD117, antibody clone AC126 (Miltenyi) and to perform qPCR. Flow cytometry was used to determine relative numbers of cells expressing c-KIT using the control steps described for FACsorting. Q-PCR was used as described above but with GAPDH as reference gene.

### Data analysis

Intensity values were determined using GeneChip Operating Software (Affymetrix) and normalised by the Robust Multiarray Average algorithm using Affymetrix Expression Console software. Statistical analysis of differential expression was conducted using the Linear Models for Microarray Data package from the Bioconductor suite in R (http://www.bioconductor.org/). The B values, *p*-values, and fold changes were used to select differentially expressed (DE) genes reaching a minimum linear expression value of 100 in all replicates of at least one sample group (*p* ≤ 0.01, fold change (FC) ≥ 2, B > 2.945). Data was normalised with ACTB and PAFAH1B2 as control genes. Principal component analysis (PCA) was conducted and displayed using Python packages. Hierarchical clustering (HC) analysis was performed using Python SciPy. Heat maps were generated using Python matplotlib.

For more sophisticated clustering analysis, we combined three advanced clustering algorithms, namely splitting merging awareness tactics (SMART) [31], binarisation of consensus partition matrix (Bi-CoPaM) [30], and Bimax [71], in the consensus clustering framework to form two stages of consensus and to produce clusters of consistently co-expressed genes with higher resolution in three datasets, i.e. adult blood, cord blood and hiPSC datasets. We clustered each dataset separately using the SMART algorithm 100 times. Then we combined the 100 results of each dataset using Bi-CoPaM in the first stage. In the second stage, we used Bimax to combine three intermediate consensus-clustering results for individual datasets and discover the genes that consistently co-express in all three datasets. SMART and Bi-CoPaM algorithms were implemented in MATLAB, and Bimax from the biclust package in R. We took the median of the replicates in each population. Therefore, we have 11 population points for adult blood (namely d0, SEM-F d4, SEM-i d4, SEM-F d7-, SEM-F d7+, SEM-F d7 BEADS, SEM-i d7 BEADS, SEM-F d10, SEM-F d14 BEADS, SEM-F d14, and SEM-i d14; for ease of visualisation to maintain an odd number of populations, a 12th, SEM-F d12, was omitted); 3 population points for cord blood (namely d0, d7 and d14); and 3 population points for hiPSC (namely d0, d7 and d14). There are no data-dependent parameters to set. The maximum number of merges in SMART is 10; the tuning parameter in Bi-CoPaM is 0, meaning that a gene is assigned to only one cluster; the parameter for the minimum number of columns is 3, which is the number of datasets, meaning that the resulting clusters must include only the genes co-expressing in all three datasets. Biopython [72] was employed to fetch 1 kb of genomic DNA sequence upstream of the transcriptional start sites (TSS) of each gene in the cluster. MEME suite was used for transcription factor binding site (TFBS) analysis, seeking homology with motifs in the Jolma database [73]. Differentially-expressed genes were examined for enriched functional ontologies using GeneCoDis [74].

## Additional files

Additional file 1: Figure S2. Erythroblast maturation and study design. A) Erythroid differentiation was induced in adult peripheral blood CD34^+^ cells in SEM-F (Table 1) from three independent samples for each population isolated. Triplicate samples of total CD34^+^ cells were also obtained on day 0 for RNA extraction. B) Fluorescence-activated cell sorting (FACS) was used to isolate triplicate samples of discrete populations of cells similar in maturity and lineage. A fraction of each sample was examined morphologically as shown using Wright stain to confirm the isolation of populations of cells which had progressively developed changes in size, nuclear-cytoplasmic ratio, cytoplasmic staining and nuclear positioning typical of terminal erythropoiesis. Scale bar equates to 10 μm. C) Cell proliferation in culture in SEM-F. Five representative samples are shown tracking fold change in total cell number over time, allowing for dilution during culturing schedules. D) Analytical workflow. Linear Models for Microarray Data was used for statistical analysis of differential expressed (DE) genes (B value at least 2.945, p-value below 0.01, fold change at least 2, and expression levels in all replicates at any population at least 100). The resulting DE genes were analysed by PCA. To show the global view of expression patterns, the DE genes were also clustered using unsupervised hierarchical clustering and displayed using heat maps. More sophisticated clustering was conducted to select consistently co-expressed genes in adult blood, cord blood, and hiPSC datasets. The consensus clustering framework combining SMART, Bi-CoPaM and Bimax is described in the Methods section. Thereafter, the GO term and upstream transcriptional factor binding site analyses of the resulting clusters were conducted. (PDF 599 kb)
Additional file 2:
**Tables S1A** and **S1B**. Both relate to Fig. 1. (XLSX 9540 kb)
Additional file 3:
**Figure S4.** Gene expression centroids from the adult FBS erythropoiesis data as depicted in Fig. 1B. Replicate samples are plotted as individual data points. Error bars indicate the standard deviation of expression observed. (PDF 130 kb)
Additional file 4: Table S2. Linear expression values of all genes from all HSC sources studied that exceed a threshold linear expression level of 100 in all replicates of at least one population. (CSV 17480 kb)
Additional file 5: Tables S3A and S3B. Both relate to Fig. 2. (XLSX 6267 kb)
Additional file 6: Figure S5A. Microarray gene expression profiles for selected genes with roles in erythropoiesis, in the AB-erythroblasts and CB-erythroblasts cultured in SEM-F. Mean expression ± standard error of the mean is plotted. (PDF 303 kb)
Additional file 7: Figure S5B. Relates to Fig. 2. Quantitative PCR validation of fold changes in gene expression observed by microarray. For each DE gene, the left hand graph shows the change in expression at each erythroid culture stage observed by microarray (“HTA”), and the right hand graph shows the same FC verified by qPCR (of 2 representative samples in quadruplicate); mean ± SD. A logarithmic scale (base 10) is used. Erythroid Stage plotted on the x-axes: d0, day 0 (CD34+); d7-, day 7- (SEM-F, CD71 + CD235a-); d7+ (SEM-F, CD71+ CD235a+) d14, day 14 (SEM-F, CD235a+). (PDF 31 kb)
Additional file 8: Figure S6. HCL analysis of the union of DE genes during AB-erythroblast maturation in SEM-F or SEM-i, as described in the manuscript. HCL was prepared by Euclidean distance clustering by gene and by sample. The colour bar on the left hand side denotes clusters of co-regulated genes. (PDF 4146 kb)
Additional file 9: Figure S7. The effect of different media types on gene expression in maturing AB-erythroblasts The numbers of genes DE between replicates of the same populations (defined by the sort gates in Additional file 1: Figure S2 and Additional file 18: Figure S3) are plotted. Those with lower expression in the first medium indicated are shown in blue; those with higher expression in red, within the ranges of fold changes indicated on the x axis for SEM-F versus SEM-i. The numbers of genes DE between AB-erythroblasts at the same stages in different media are insignificant compared with the large number of genes expressed in AB-erythroblasts at any one stage. (PDF 44 kb)
Additional file 10: Tables S5A and S5B. Both relate to Fig. 4 and contain annotations, linear expression values, statistical parameters and fold changes of differentially expressed genes between different populations during maturation of Adult and hiPSC Erythroblasts in SEM-i. Inclusion criteria for differentially expressed genes: B value of at least 2.945, *p*-value below 0.01, fold change of at least 2, and expression levels in all replicates at any population of at least 100. Erythroblasts were cultured for between 0 and 14 days (d0-d14). Upregulated and downregulated genes are listed separately in Table S5B. (XLSX 5326 kb)
Additional file 11: Figure S8. Gene expression profiles for selected genes with roles in heme biosynthesis, in AB-erythroblasts and hiPSC-erythroblasts cultured in SEM-i. Mean expression +/- standard error of the mean is plotted. (PDF 295 kb)
Additional file 12: Figure S9. A) Gene ontology analysis of genes regulated during the first 7 days of culture of AB-erythroblasts and hiPSC-erythroblasts in SEM-i. Significantly-scoring ontologies are shown, together with the more stringent corrected hypergeometric *p*-values produced by GeneCoDis, and the number of DE genes in each category from both cell types. B) Numbers of genes with roles in the cell cycle which are up-regulated in AB-erythroblasts and hiPSC-erythroblasts in SEM-i between days 0 and 7 are indicated, showing the numbers up-regulated from both sources or uniquely in each. (PDF 408 kb)
Additional file 13: Figure S10B. Quantitative PCR validation of fold changes of selected genes observed by microarray. For each DE gene, the left hand graph shows fold changes (FC) relative to AB day 0 observed by microarray (“HTA”), and the right hand graph shows the same FC verified by QPCR (2 repeats of 2 representative samples in triplicate). A logarithmic scale (base 10) is used. Time is plotted on the x-axes: d0, day 0 (CD34+); d7, day 7 (SEM-i, CD71+); d14, day 14 (SEM-i, CD235a+). (PDF 383 kb)
Additional file 14: Figure S10A. Gene expression profiles for selected genes with roles in the control of erythropoiesis, in AB-erythroblasts and hiPSC-erythroblasts cultured in SEM-i. Mean expression ± standard error of the mean is plotted. (PDF 298 kb)
Additional file 15: Figure S11. Expression profiles of genes within all clusters of >30 members derived from the SMART analysis of the global dataset are shown. Top row AB-erythroblasts, middle row CB-erythroblasts, bottom row hiPSC-erythroblasts. (PDF 287 kb)
Additional file 16: Figure S13. HCL analysis of the union of DE genes from all cells of origin, in all media as described in the manuscript. HCL was prepared by Euclidean distance clustering by gene and by sample. A denotes adult, C denotes cord blood, and iPS denotes hiPSC-derived erythroid cultures. Samples isolated using paramagnetic beads rather than flow sorting are labelled. The colour bar on the left hand side denotes clusters of co-regulated genes. (PDF 5324 kb)
Additional file 17: Figure S1. The cell surface phenotype of progenitors derived from hiPSC co-culture on OP9 stromal cells. The proportion of cells that express cell surface proteins used to identify hematopoietic progenitors are shown in (A) on day 0 before transfer to SEM-i culture where (i) the CD31+ gate comprises more than 90 % CD34+ when CD43+ and CD43- are combined and (ii) only 3 % of CD34 + CD31- cells express the hematopoietic lineage marker CD43. On day 3 shown in (B) a Percoll gradient has removed OP9 cells so that (i) the viable DAPI negative culture grown on is (ii) enriched with approximately 98 % CD43+ hematopoietic cells and 30 % of cells are typically CD34+ some of which also express CD45. This indicates enrichment of hematopoietic multipotent progenitors during the first phase of culture in SEM-i as described previously by Dias et al. [18]. (PDF 144 kb)
Additional file 18: Figure S3. Isolation of erythroid precursors at defined stages of culture and derived from different sources of hematopoietic progenitors. A) Eryhthroblasts derived from cord blood CD34+ cells and cultured in SEM-F were enriched by FACs and gene expression compared to that from adult derived erythroblasts isolated using the same sort gates shown in Figure S2. The CD markers expressed by erythroblasts in the collected fractions are highlighted in blue. B) Erythroblast numbers derived from hIPSC progenitors grown in SEM-i were not sufficient for FACSorting and so populations based on time and phenotype were isolated using magnetic bead isolation. Day 7 erythroblasts were isolated using CD71 specific beads to target early precursors and Day 14 erythroblasts were isolated using beads specific to CD235a to target more mature erythroid cells. C) For comparison with hiPSC, CD34+ cells from Adult peripheral blood were cultured in the same media (SEM-i) and magnetic beads specific to CD71+ and CD235a + also used to isolate the populations shown on days 7 and 14 respectively. An example of sort purity achieved after D) use of FACS to obtain CD235a + CD71+ erythroblasts after 14 days of Adult HSC culture in SEM-F. The R5 sort gate was used to collect erythroblasts that were 94 % pure based on the expression of both CD235a and CD71. E) Magnetic beads conjugated to antibodies specific to CD71 were used on day 7 and beads specific to CD235a were used on day 14 of erythroid cultures in SEM-i. The purity of cells collected shown in R3 gate on day 7 or in R4 gate on day 14 is highlighted in blue. (PDF 190 kb)
Additional file 19:
**Tables S6**, **S7** and **S8.** The Euclidean distances from the PCAs shown in Figs. 1, 2 and 4 of manuscript. (PDF 96 kb)
Additional file 20: Table S4. Relates to Fig. 3. Annotations, linear expression values, statistical parameters and fold changes of genes DE (B value at least 2.945, *p*-value below 0.01, fold change at least 2, and expression levels in all replicates at any population at least 100) between different populations during maturation of Adult Erythroblasts (AB-erythroblasts) in SEM-F or SEM-i, cultured for between 0 and 14 days (d0-d14). (XLSX 10526 kb)
Additional file 21: Figure S12. Enucleated cells were enumerated from stained cytospin preparations prepared after extended culture period of 21 days in SEM-F. Samples were analysed before and after CD235a selection on paramagnetic beads. Observations from 2 independent cultures in SEM-i are shown from a minimum of 90 cells per culture condition. (PDF 21 kb)

## Abbreviations

AB: Adult peripheral blood
Bi-CoPaM: Binarisation of consensus partition matrix
CB: Cord blood
DE: Differentially expressed
EPO: Erythropoietin
FACS: Fluorescence activated cell sorting
GYPA/CD235a: Glycophorin A
HC: Hierarchical clustering
HiPSC: Human induced pluripotent stem cells
HSC: Hematopoietic stem cell
HSPC: Hematopoietic stem/progenitor cells
KLF: Kruppel-like factor erythroid
MEP: Megakaryocyte/erythroid potential
PCA: Principal component analyses
RBCs: Red blood cells
SCF: Stem cell factor
SEM: Standard erythroid media
SMART: Splitting merging awareness tactics
TF: Transcription factor
TFBS: Transcription factor binding site
TSS: Transcriptional start sites

## References

[1] Kaufman DS, Hanson ET, Lewis RL, Auerbach R, Thomson JA (2001). Hematopoietic colony-forming cells derived from human embryonic stem cells. Proc Natl Acad Sci U S A.

[2] Zambidis ET, Peault B, Park TS, Bunz F, Civin CI (2005). Hematopoietic differentiation of human embryonic stem cells progresses through sequential hematoendothelial, primitive, and definitive stages resembling human yolk sac development. Blood.

[3] Baron MH (2013). Concise Review: early embryonic erythropoiesis: not so primitive after all. Stem Cells.

[4] McGrath KE, Kingsley PD, Koniski AD, Porter RL, Bushnell TP, Palis J (2008). Enucleation of primitive erythroid cells generates a transient population of “pyrenocytes” in the mammalian fetus. Blood.

[5] Grover A, Mancini E, Moore S, Mead AJ, Atkinson D, Rasmussen KD, O’Carroll D, Jacobsen SE, Nerlov C (2014). Erythropoietin guides multipotent hematopoietic progenitor cells toward an erythroid fate. J Exp Med.

[6] Chou ST, Khandros E, Bailey LC, Nichols KE, Vakoc CR, Yao Y, Huang Z, Crispino JD, Hardison RC, Blobel GA (2009). Graded repression of PU.1/Sfpi1 gene transcription by GATA factors regulates hematopoietic cell fate. Blood.

[7] Wontakal SN, Guo X, Smith C, MacCarthy T, Bresnick EH, Bergman A, Snyder MP, Weissman SM, Zheng D, Skoultchi AI (2012). A core erythroid transcriptional network is repressed by a master regulator of myelo-lymphoid differentiation. Proc Natl Acad Sci U S A.

[8] Wu W, Cheng Y, Keller CA, Ernst J, Kumar SA, Mishra T, Morrissey C, Dorman CM, Chen KB, Drautz D (2011). Dynamics of the epigenetic landscape during erythroid differentiation after GATA1 restoration. Genome Res.

[9] Giarratana MC, Kobari L, Lapillonne H, Chalmers D, Kiger L, Cynober T, Marden MC, Wajcman H, Douay L (2005). Ex vivo generation of fully mature human red blood cells from hematopoietic stem cells. Nat Biotechnol.

[10] Griffiths RE, Kupzig S, Cogan N, Mankelow TJ, Betin VM, Trakarnsanga K, Massey EJ, Lane JD, Parsons SF, Anstee DJ (2012). Maturing reticulocytes internalize plasma membrane in glycophorin A-containing vesicles that fuse with autophagosomes before exocytosis. Blood.

[11] Chang CJ, Mitra K, Koya M, Velho M, Desprat R, Lenz J, Bouhassira EE (2011). Production of embryonic and fetal-like red blood cells from human induced pluripotent stem cells. PLoS One.

[12] Lapillonne H, Kobari L, Mazurier C, Tropel P, Giarratana MC, Zanella-Cleon I, Kiger L, Wattenhofer-Donze M, Puccio H, Hebert N (2010). Red blood cell generation from human induced pluripotent stem cells: perspectives for transfusion medicine. Haematologica.

[13] Lu SJ, Feng Q, Park JS, Vida L, Lee BS, Strausbauch M, Wettstein PJ, Honig GR, Lanza R (2008). Biologic properties and enucleation of red blood cells from human embryonic stem cells. Blood.

[14] Yang CT, French A, Goh PA, Pagnamenta A, Mettananda S, Taylor J, Knight S, Nathwani A, Roberts DJ, Watt SM (2014). Human induced pluripotent stem cell derived erythroblasts can undergo definitive erythropoiesis and co-express gamma and beta globins. Br J Haematol.

[15] Byrska-Bishop M, VanDorn D, Campbell AE, Betensky M, Arca PR, Yao Y, Gadue P, Costa FF, Nemiroff RL, Blobel GA (2015). Pluripotent stem cells reveal erythroid-specific activities of the GATA1 N-terminus. J Clin Invest.

[16] Chang CJ, Bouhassira EE (2012). Zinc-finger nuclease-mediated correction of alpha-thalassemia in iPS cells. Blood.

[17] Chou ST, Byrska-Bishop M, Tober JM, Yao Y, Vandorn D, Opalinska JB, Mills JA, Choi JK, Speck NA, Gadue P (2012). Trisomy 21-associated defects in human primitive hematopoiesis revealed through induced pluripotent stem cells. Proc Natl Acad Sci U S A.

[18] Dias J, Gumenyuk M, Kang H, Vodyanik M, Yu J, Thomson JA, Slukvin II (2011). Generation of red blood cells from human induced pluripotent stem cells. Stem Cells Dev.

[19] Hanna J, Wernig M, Markoulaki S, Sun CW, Meissner A, Cassady JP, Beard C, Brambrink T, Wu LC, Townes TM (2007). Treatment of sickle cell anemia mouse model with iPS cells generated from autologous skin. Science.

[20] Huang X, Wang Y, Yan W, Smith C, Ye Z, Wang J, Gao Y, Mendelsohn L, Cheng L (2015). Production of gene-corrected adult beta globin protein in human erythrocytes differentiated from patient iPSCs after genome editing of the sickle point mutation. Stem Cells.

[21] Mazurier C, Douay L, Lapillonne H (2011). Red blood cells from induced pluripotent stem cells: hurdles and developments. Curr Opin Hematol.

[22] Salvagiotto G, Burton S, Daigh CA, Rajesh D, Slukvin II, Seay NJ (2011). A defined, feeder-free, serum-free system to generate in vitro hematopoietic progenitors and differentiated blood cells from hESCs and hiPSCs. PLoS One.

[23] Trakarnsanga K, Wilson MC, Griffiths RE, Toye AM, Carpenter L, Heesom KJ, Parsons SF, Anstee DJ, Frayne J (2014). Qualitative and quantitative comparison of the proteome of erythroid cells differentiated from human iPSCs and adult erythroid cells by multiplex TMT labelling and NanoLC-MS/MS. PLoS One.

[24] Kobari L, Yates F, Oudrhiri N, Francina A, Kiger L, Mazurier C, Rouzbeh S, El-Nemer W, Hebert N, Giarratana MC (2012). Human induced pluripotent stem cells can reach complete terminal maturation: in vivo and in vitro evidence in the erythropoietic differentiation model. Haematologica.

[25] Merryweather-Clarke AT, Atzberger A, Soneji S, Gray N, Clark K, Waugh C, McGowan SJ, Taylor S, Nandi AK, Wood WG (2011). Global gene expression analysis of human erythroid progenitors. Blood.

[26] Keller MA, Addya S, Vadigepalli R, Banini B, Delgrosso K, Huang H, Surrey S (2006). Transcriptional regulatory network analysis of developing human erythroid progenitors reveals patterns of coregulation and potential transcriptional regulators. Physiol Genomics.

[27] An X, Schulz VP, Li J, Wu K, Liu J, Xue F, Hu J, Mohandas N, Gallagher PG (2014). Global transcriptome analyses of human and murine terminal erythroid differentiation. Blood.

[28] Li B, Ding L, Yang C, Kang B, Liu L, Story MD, Pace BS (2014). Characterization of transcription factor networks involved in umbilical cord blood CD34+ stem cells-derived erythropoiesis. PLoS One.

[29] Li J, Hale J, Bhagia P, Xue F, Chen L, Jaffray J, Yan H, Lane J, Gallagher PG, Mohandas N (2014). Isolation and transcriptome analyses of human erythroid progenitors: BFU-E and CFU-E. Blood.

[30] Abu-Jamous B, Fa R, Roberts DJ, Nandi AK (2013). Paradigm of tunable clustering using Binarization of Consensus Partition Matrices (Bi-CoPaM) for gene discovery. PLoS One.

[31] Fa R, Roberts DJ, Nandi AK (2014). SMART: unique splitting-while-merging framework for gene clustering. PLoS One.

[32] Novershtern N, Subramanian A, Lawton LN, Mak RH, Haining WN, McConkey ME, Habib N, Yosef N, Chang CY, Shay T (2011). Densely interconnected transcriptional circuits control cell states in human hematopoiesis. Cell.

[33] Chang KH, Nelson AM, Cao H, Wang L, Nakamoto B, Ware CB, Papayannopoulou T (2006). Definitive-like erythroid cells derived from human embryonic stem cells coexpress high levels of embryonic and fetal globins with little or no adult globin. Blood.

[34] Palis J (2014). Primitive and definitive erythropoiesis in mammals. Front Physiol.

[35] Sankaran VG, Xu J, Ragoczy T, Ippolito GC, Walkley CR, Maika SD, Fujiwara Y, Ito M, Groudine M, Bender MA (2009). Developmental and species-divergent globin switching are driven by BCL11A. Nature.

[36] Xu J, Sankaran VG, Ni M, Menne TF, Puram RV, Kim W, Orkin SH (2010). Transcriptional silencing of gamma-globin by BCL11A involves long-range interactions and cooperation with SOX6. Genes Dev.

[37] Yi Z, Cohen-Barak O, Hagiwara N, Kingsley PD, Fuchs DA, Erickson DT, Epner EM, Palis J, Brilliant MH (2006). Sox6 directly silences epsilon globin expression in definitive erythropoiesis. PLoS Genet.

[38] Kingsley PD, Greenfest-Allen E, Frame JM, Bushnell TP, Malik J, McGrath KE, Stoeckert CJ, Palis J (2013). Ontogeny of erythroid gene expression. Blood.

[39] Starnes LM, Sorrentino A, Ferracin M, Negrini M, Pelosi E, Nervi C, Peschle C (2010). A transcriptome-wide approach reveals the key contribution of NFI-A in promoting erythroid differentiation of human CD34(+) progenitors and CML cells. Leukemia.

[40] Betin VM, Singleton BK, Parsons SF, Anstee DJ, Lane JD (2013). Autophagy facilitates organelle clearance during differentiation of human erythroblasts: evidence for a role for ATG4 paralogs during autophagosome maturation. Autophagy.

[41] Zhang J, Randall MS, Loyd MR, Dorsey FC, Kundu M, Cleveland JL, Ney PA (2009). Mitochondrial clearance is regulated by Atg7-dependent and -independent mechanisms during reticulocyte maturation. Blood.

[42] Ju JS, Weihl CC (2010). p97/VCP at the intersection of the autophagy and the ubiquitin proteasome system. Autophagy.

[43] Ramadan K, Bruderer R, Spiga FM, Popp O, Baur T, Gotta M, Meyer HH (2007). Cdc48/p97 promotes reformation of the nucleus by extracting the kinase Aurora B from chromatin. Nature.

[44] Thom CS, Traxler EA, Khandros E, Nickas JM, Zhou OY, Lazarus JE, Silva AP, Prabhu D, Yao Y, Aribeana C (2014). Trim58 degrades Dynein and regulates terminal erythropoiesis. Dev Cell.

[45] Yang Y, Wang H, Chang KH, Qu H, Zhang Z, Xiong Q, Qi H, Cui P, Lin Q, Ruan X (2013). Transcriptome dynamics during human erythroid differentiation and development. Genomics.

[46] Xu J, Shao Z, Glass K, Bauer DE, Pinello L, Van Handel B, Hou S, Stamatoyannopoulos JA, Mikkola HK, Yuan GC (2012). Combinatorial assembly of developmental stage-specific enhancers controls gene expression programs during human erythropoiesis. Dev Cell.

[47] Bell AJ, Satchwell TJ, Heesom KJ, Hawley BR, Kupzig S, Hazell M, Mushens R, Herman A, Toye AM (2013). Protein distribution during human erythroblast enucleation in vitro. PLoS One.

[48] Keerthivasan G, Wickrema A, Crispino JD (2011). Erythroblast enucleation. Stem Cells Int.

[49] Kadri Z, Maouche-Chretien L, Rooke HM, Orkin SH, Romeo PH, Mayeux P, Leboulch P, Chretien S (2005). Phosphatidylinositol 3-kinase/Akt induced by erythropoietin renders the erythroid differentiation factor GATA-1 competent for TIMP-1 gene transactivation. Mol Cell Biol.

[50] Tallack MR, Magor GW, Dartigues B, Sun L, Huang S, Fittock JM, Fry SV, Glazov EA, Bailey TL, Perkins AC (2012). Novel roles for KLF1 in erythropoiesis revealed by mRNA-seq. Genome Res.

[51] Lieu YK, Reddy EP (2009). Conditional c-myb knockout in adult hematopoietic stem cells leads to loss of self-renewal due to impaired proliferation and accelerated differentiation. Proc Natl Acad Sci U S A.

[52] Bauer DE, Orkin SH (2015). Hemoglobin switching’s surprise: the versatile transcription factor BCL11A is a master repressor of fetal hemoglobin. Curr Opin Genet Dev.

[53] Sadahira Y, Yoshino T, Monobe Y (1995). Very late activation antigen 4-vascular cell adhesion molecule 1 interaction is involved in the formation of erythroblastic islands. J Exp Med.

[54] Douay L, Giarratana MC (2009). Ex vivo generation of human red blood cells: a new advance in stem cell engineering. Methods Mol Biol.

[55] Doulatov S, Vo LT, Chou SS, Kim PG, Arora N, Li H, Hadland BK, Bernstein ID, Collins JJ, Zon LI (2013). Induction of multipotential hematopoietic progenitors from human pluripotent stem cells via respecification of lineage-restricted precursors. Cell Stem Cell.

[56] Dorn I, Klich K, Arauzo-Bravo MJ, Radstaak M, Santourlidis S, Ghanjati F, Radke TF, Psathaki OE, Hargus G, Kramer J (2015). Erythroid differentiation of human induced pluripotent stem cells is independent of donor cell type of origin. Haematologica.

[57] Sturgeon CM, Ditadi A, Awong G, Kennedy M, Keller G (2014). Wnt signaling controls the specification of definitive and primitive hematopoiesis from human pluripotent stem cells. Nat Biotechnol.

[58] Clevers H, Loh KM, Nusse R (2014). Stem cell signaling. An integral program for tissue renewal and regeneration: Wnt signaling and stem cell control. Science.

[59] Trowbridge JJ, Xenocostas A, Moon RT, Bhatia M (2006). Glycogen synthase kinase-3 is an in vivo regulator of hematopoietic stem cell repopulation. Nat Med.

[60] Ploper D, De Robertis EM (2015). The MITF family of transcription factors: Role in endolysosomal biogenesis, Wnt signaling, and oncogenesis. Pharmacol Res.

[61] Isern J, He Z, Fraser ST, Nowotschin S, Ferrer-Vaquer A, Moore R, Hadjantonakis AK, Schulz V, Tuck D, Gallagher PG (2011). Single-lineage transcriptome analysis reveals key regulatory pathways in primitive erythroid progenitors in the mouse embryo. Blood.

[62] Felli N, Fontana L, Pelosi E, Botta R, Bonci D, Facchiano F, Liuzzi F, Lulli V, Morsilli O, Santoro S (2005). MicroRNAs 221 and 222 inhibit normal erythropoiesis and erythroleukemic cell growth via kit receptor down-modulation. Proc Natl Acad Sci U S A.

[63] Kajiguchi T, Lee S, Lee MJ, Trepel JB, Neckers L (2008). KIT regulates tyrosine phosphorylation and nuclear localization of beta-catenin in mast cell leukemia. Leuk Res.

[64] Shtutman M, Zhurinsky J, Simcha I, Albanese C, D'Amico M, Pestell R, Ben-Ze’ev A (1999). The cyclin D1 gene is a target of the beta-catenin/LEF-1 pathway. Proc Natl Acad Sci U S A.

[65] Horne WC, Huang SC, Becker PS, Tang TK, Benz EJ (1993). Tissue-specific alternative splicing of protein 4.1 inserts an exon necessary for formation of the ternary complex with erythrocyte spectrin and F-actin. Blood.

[66] Yamamoto ML, Clark TA, Gee SL, Kang JA, Schweitzer AC, Wickrema A, Conboy JG (2009). Alternative pre-mRNA splicing switches modulate gene expression in late erythropoiesis. Blood.

[67] Pimentel H, Parra M, Gee S, Ghanem D, An X, Li J, Mohandas N, Pachter L, Conboy JG (2014). A dynamic alternative splicing program regulates gene expression during terminal erythropoiesis. Nucleic Acids Res.

[68] Shi L, Lin YH, Sierant MC, Zhu F, Cui S, Guan Y, Sartor MA, Tanabe O, Lim KC, Engel JD (2014). Developmental transcriptome analysis of human erythropoiesis. Hum Mol Genet.

[69] Griffiths RE, Kupzig S, Cogan N, Mankelow TJ, Betin VM, Trakarnsanga K, Massey EJ, Parsons SF, Anstee DJ, Lane JD (2012). The ins and outs of human reticulocyte maturation: autophagy and the endosome/exosome pathway. Autophagy.

[70] Demaison C, Parsley K, Brouns G, Scherr M, Battmer K, Kinnon C, Grez M, Thrasher AJ (2002). High-level transduction and gene expression in hematopoietic repopulating cells using a human immunodeficiency [correction of imunodeficiency] virus type 1-based lentiviral vector containing an internal spleen focus forming virus promoter. Hum Gene Ther.

[71] Prelic A, Bleuler S, Zimmermann P, Wille A, Buhlmann P, Gruissem W, Hennig L, Thiele L, Zitzler E (2006). A systematic comparison and evaluation of biclustering methods for gene expression data. Bioinformatics.

[72] Cock PJ, Antao T, Chang JT, Chapman BA, Cox CJ, Dalke A, Friedberg I, Hamelryck T, Kauff F, Wilczynski B (2009). Biopython: freely available Python tools for computational molecular biology and bioinformatics. Bioinformatics.

[73] Jolma A, Yan J, Whitington T, Toivonen J, Nitta KR, Rastas P, Morgunova E, Enge M, Taipale M, Wei G (2013). DNA-binding specificities of human transcription factors. Cell.

[74] Tabas-Madrid D, Nogales-Cadenas R, Pascual-Montano A (2012). GeneCodis3: a non-redundant and modular enrichment analysis tool for functional genomics. Nucleic Acids Res.

