# Supplementary figures and images for: Distinct gene expression program dynamics during erythropoiesis from human induced pluripotent stem cells compared with adult and cord blood progenitors
Source: BMC Genomics. 2016 Oct 21;17:817. doi: 10.1186/s12864-016-3134-z (PMC5073849; doi:10.1186/s12864-016-3134-z)

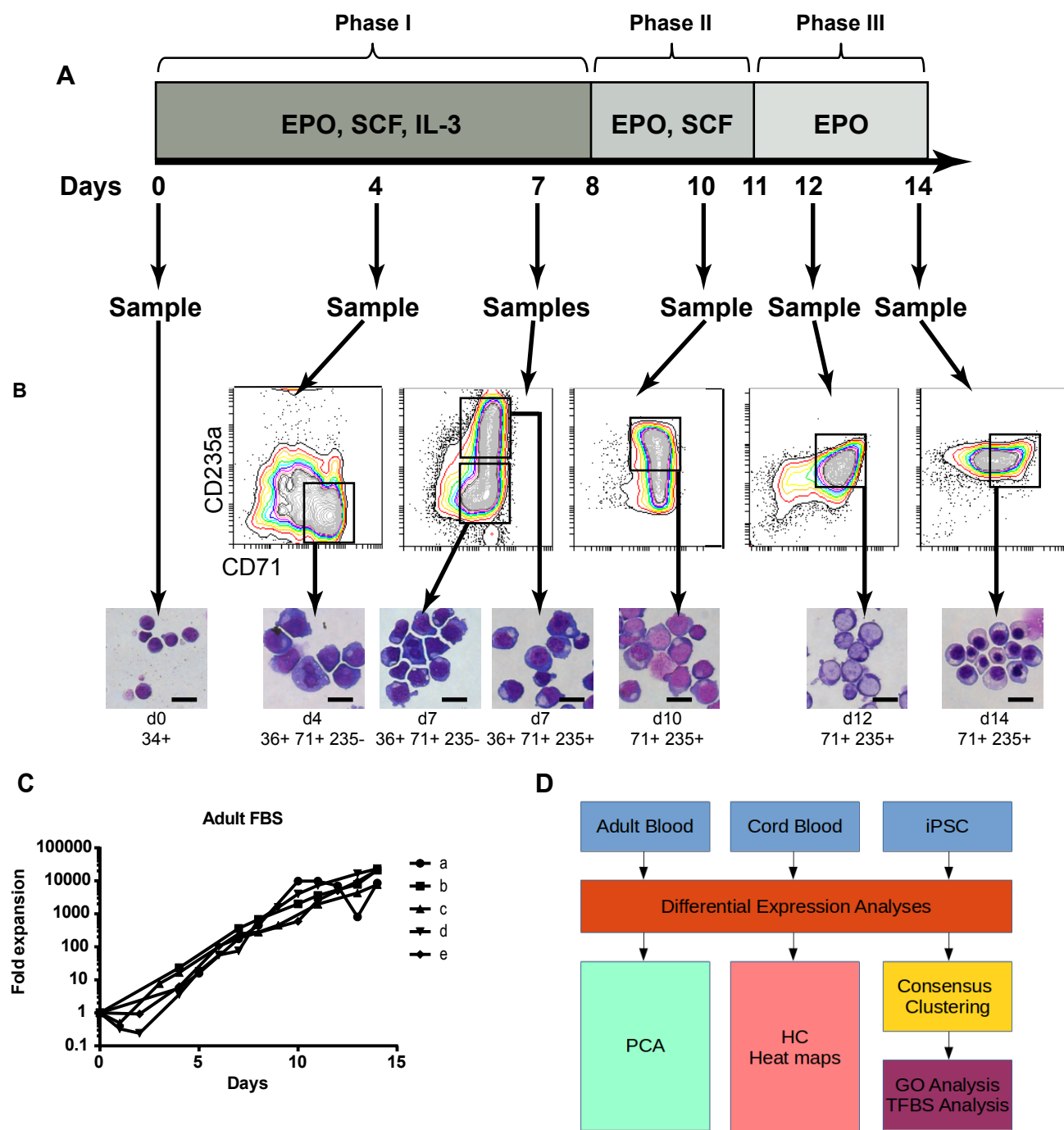

**FIGURE S2**

Supplement: Additional file 1: Figure S2. — Erythroblast maturation and study design. A) Erythroid differentiation was induced in adult peripheral blood CD34+ cells in SEM-F (Table 1) from three independent samples for each population isolated. Triplicate samples of total CD34+ cells were also obtained on day 0 for RNA extraction. B) Fluorescence-activated cell sorting (FACS) was used to isolate triplicate samples of discrete populations of cells similar in maturity and lineage. A fraction of each sample was examined morphologically as shown using Wright stain to confirm the isolation of populations of cells which had progressively developed changes in size, nuclear-cytoplasmic ratio, cytoplasmic staining and nuclear positioning typical of terminal erythropoiesis. Scale bar equates to 10 μm. C) Cell proliferation in culture in SEM-F. Five representative samples are shown tracking fold change in total cell number over time, allowing for dilution during culturing schedules. D) Analytical workflow. Linear Models for Microarray Data was used for statistical analysis of differential expressed (DE) genes (B value at least 2.945, p-value below 0.01, fold change at least 2, and expression levels in all replicates at any population at least 100). The resulting DE genes were analysed by PCA. To show the global view of expression patterns, the DE genes were also clustered using unsupervised hierarchical clustering and displayed using heat maps. More sophisticated clustering was conducted to select consistently co-expressed genes in adult blood, cord blood, and hiPSC datasets. The consensus clustering framework combining SMART, Bi-CoPaM and Bimax is described in the Methods section. Thereafter, the GO term and upstream transcriptional factor binding site analyses of the resulting clusters were conducted. (PDF 599 kb) [file 12864_2016_3134_MOESM1_ESM.pdf]

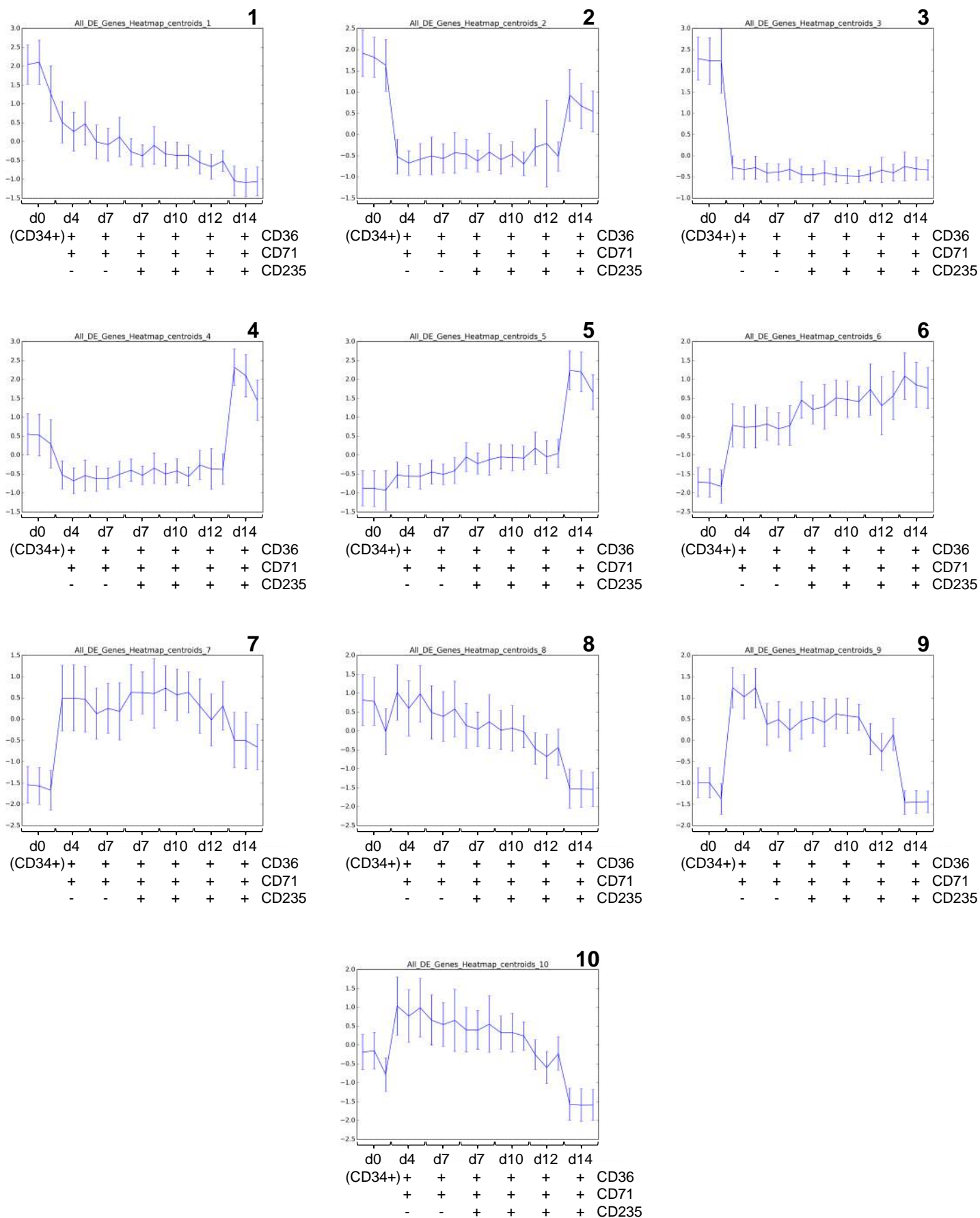

**FIGURE S4**

Supplement: Additional file 3: — Figure S4. Gene expression centroids from the adult FBS erythropoiesis data as depicted in Fig. 1B. Replicate samples are plotted as individual data points. Error bars indicate the standard deviation of expression observed. (PDF 130 kb) [file 12864_2016_3134_MOESM3_ESM.pdf]

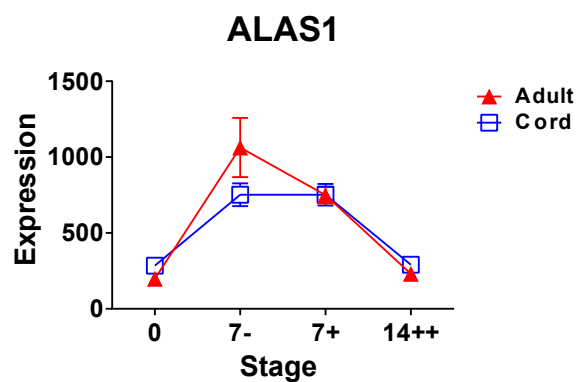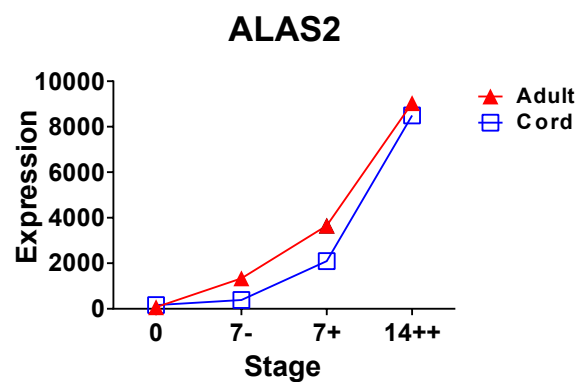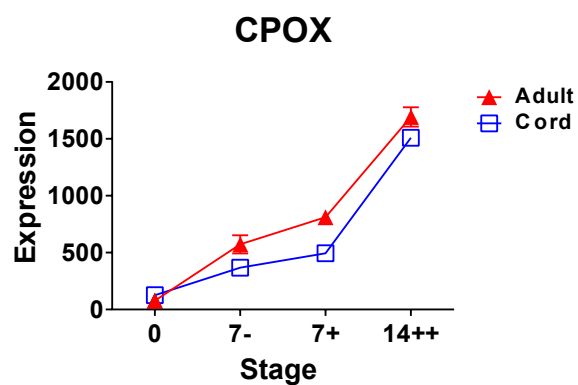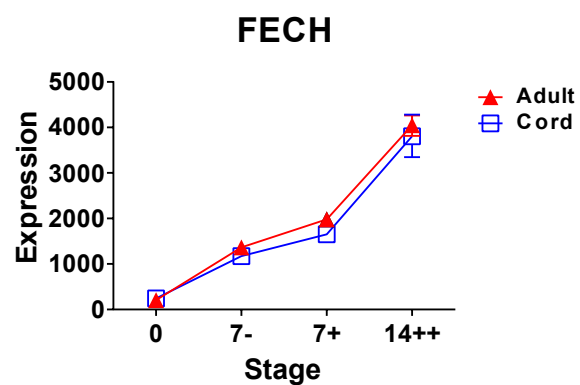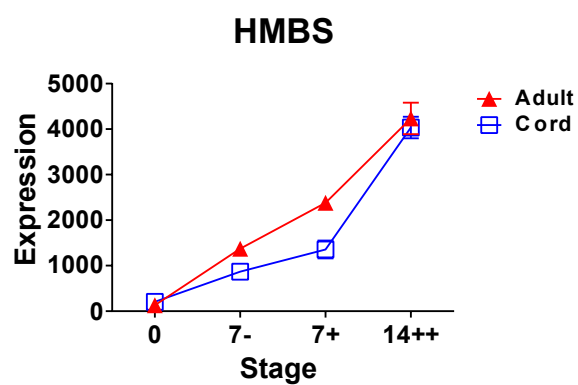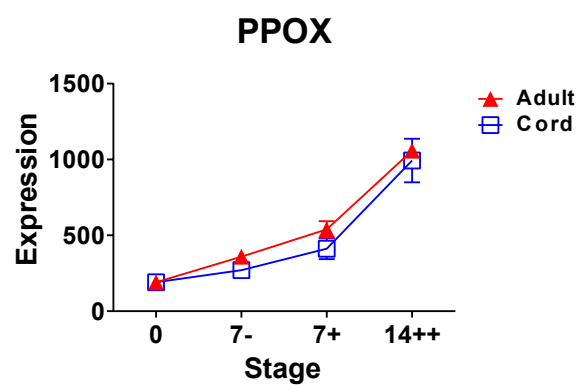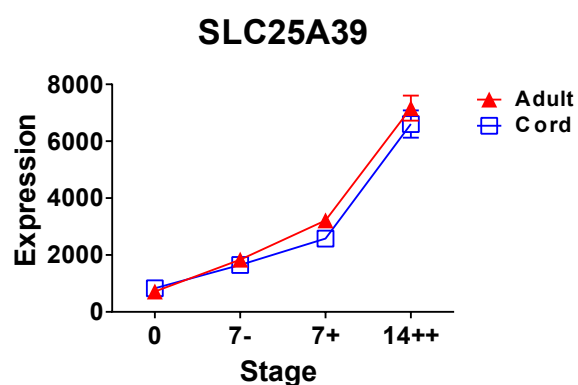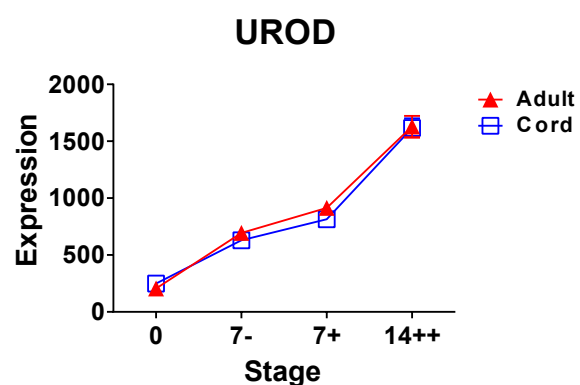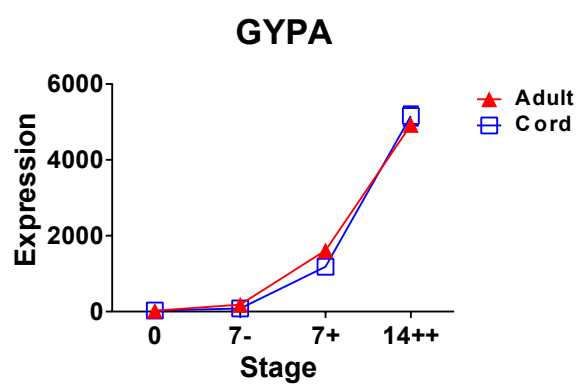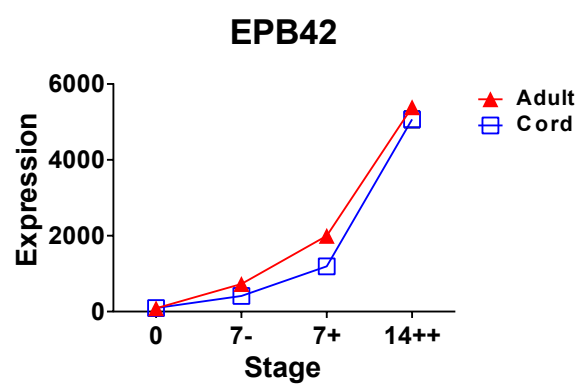

FIGURE S5A

Supplement: Additional file 6: Figure S5A. — Microarray gene expression profiles for selected genes with roles in erythropoiesis, in the AB-erythroblasts and CB-erythroblasts cultured in SEM-F. Mean expression ± standard error of the mean is plotted. (PDF 303 kb) [file 12864_2016_3134_MOESM6_ESM.pdf]

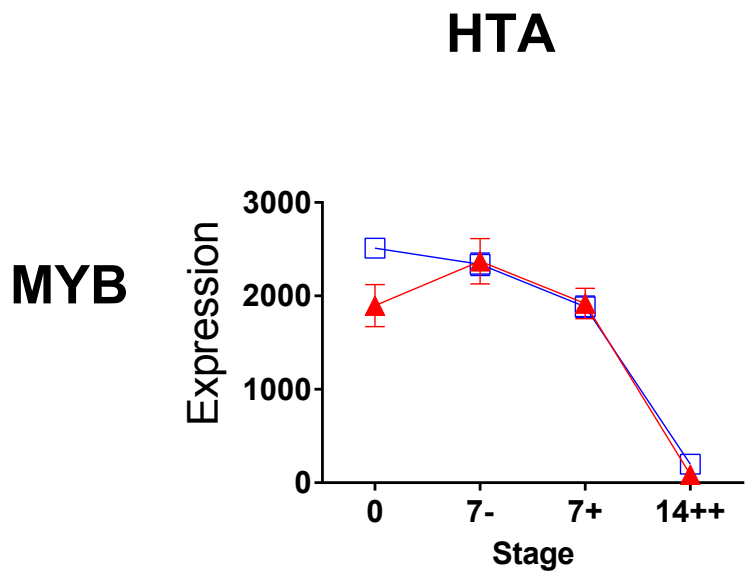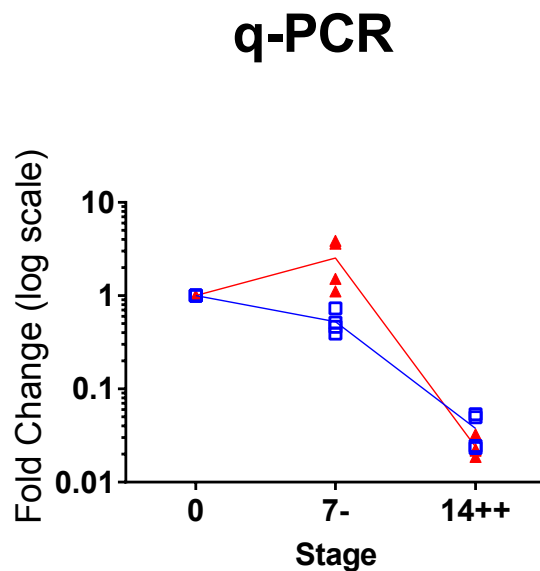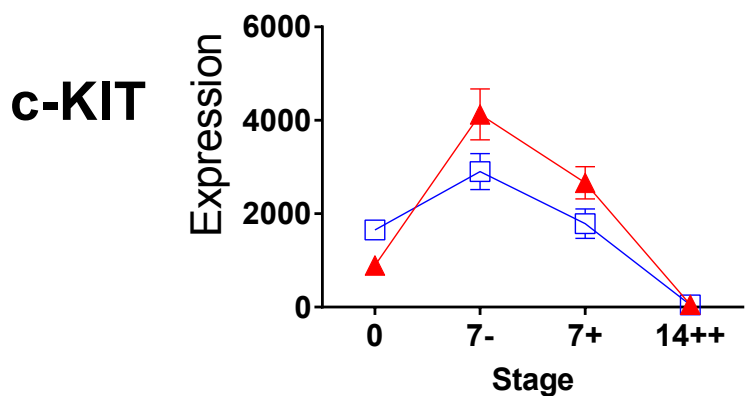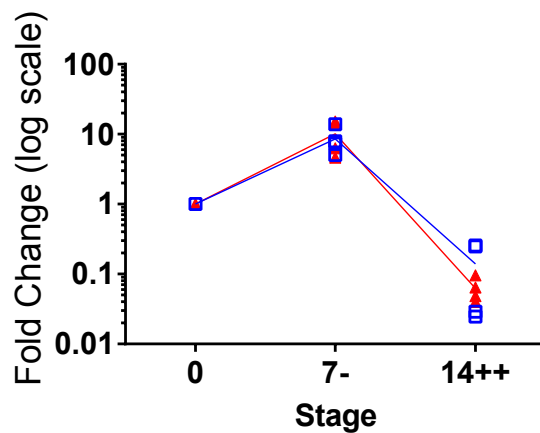

▲ Adult  
■ Cord

**Figure S5B**

Supplement: Additional file 7: Figure S5B. — Relates to Fig. 2. Quantitative PCR validation of fold changes in gene expression observed by microarray. For each DE gene, the left hand graph shows the change in expression at each erythroid culture stage observed by microarray (“HTA”), and the right hand graph shows the same FC verified by qPCR (of 2 representative samples in quadruplicate); mean ± SD. A logarithmic scale (base 10) is used. Erythroid Stage plotted on the x-axes: d0, day 0 (CD34+); d7-, day 7- (SEM-F, CD71 + CD235a-); d7+ (SEM-F, CD71+ CD235a+) d14, day 14 (SEM-F, CD235a+). (PDF 31 kb) [file 12864_2016_3134_MOESM7_ESM.pdf]

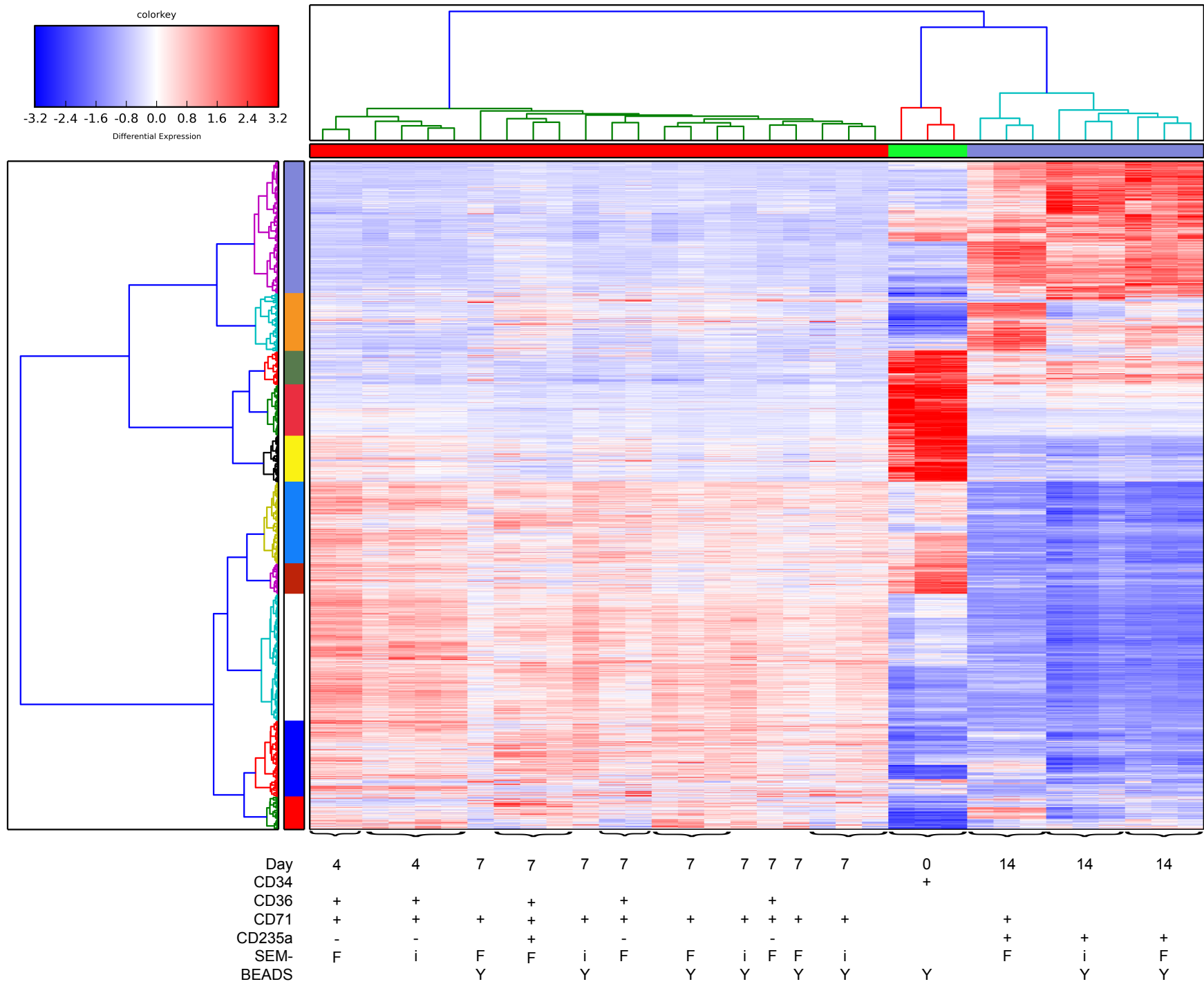

**FIGURE S6**

Supplement: Additional file 8: Figure S6. — HCL analysis of the union of DE genes during AB-erythroblast maturation in SEM-F or SEM-i, as described in the manuscript. HCL was prepared by Euclidean distance clustering by gene and by sample. The colour bar on the left hand side denotes clusters of co-regulated genes. (PDF 4146 kb) [file 12864_2016_3134_MOESM8_ESM.pdf]

## Adult-EBs, Media Effects

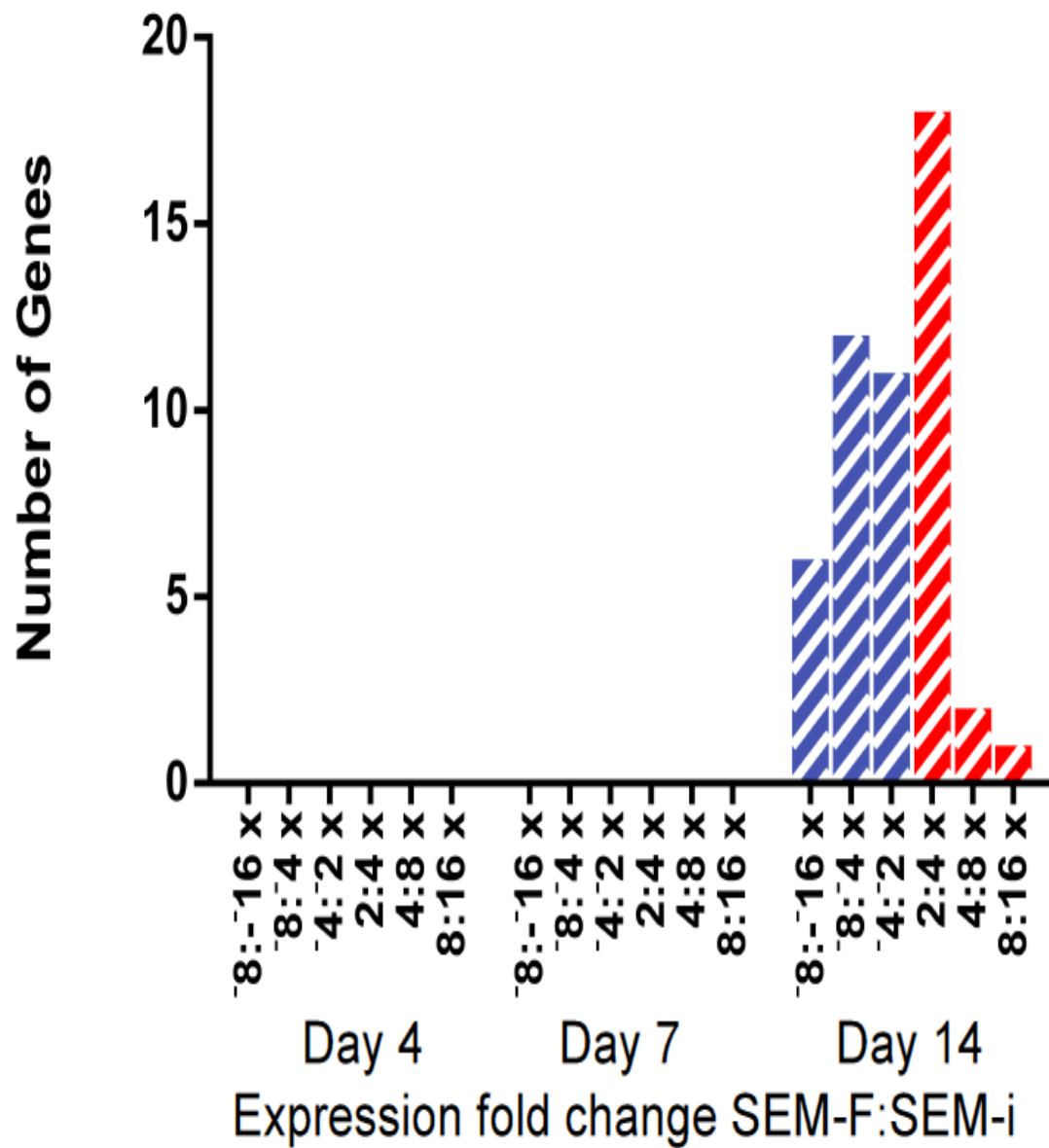

FIGURE S7

Supplement: Additional file 9: Figure S7. — The effect of different media types on gene expression in maturing AB-erythroblasts The numbers of genes DE between replicates of the same populations (defined by the sort gates in Additional file 1: Figure S2 and Additional file 18: Figure S3) are plotted. Those with lower expression in the first medium indicated are shown in blue; those with higher expression in red, within the ranges of fold changes indicated on the x axis for SEM-F versus SEM-i. The numbers of genes DE between AB-erythroblasts at the same stages in different media are insignificant compared with the large number of genes expressed in AB-erythroblasts at any one stage. (PDF 44 kb) [file 12864_2016_3134_MOESM9_ESM.pdf]

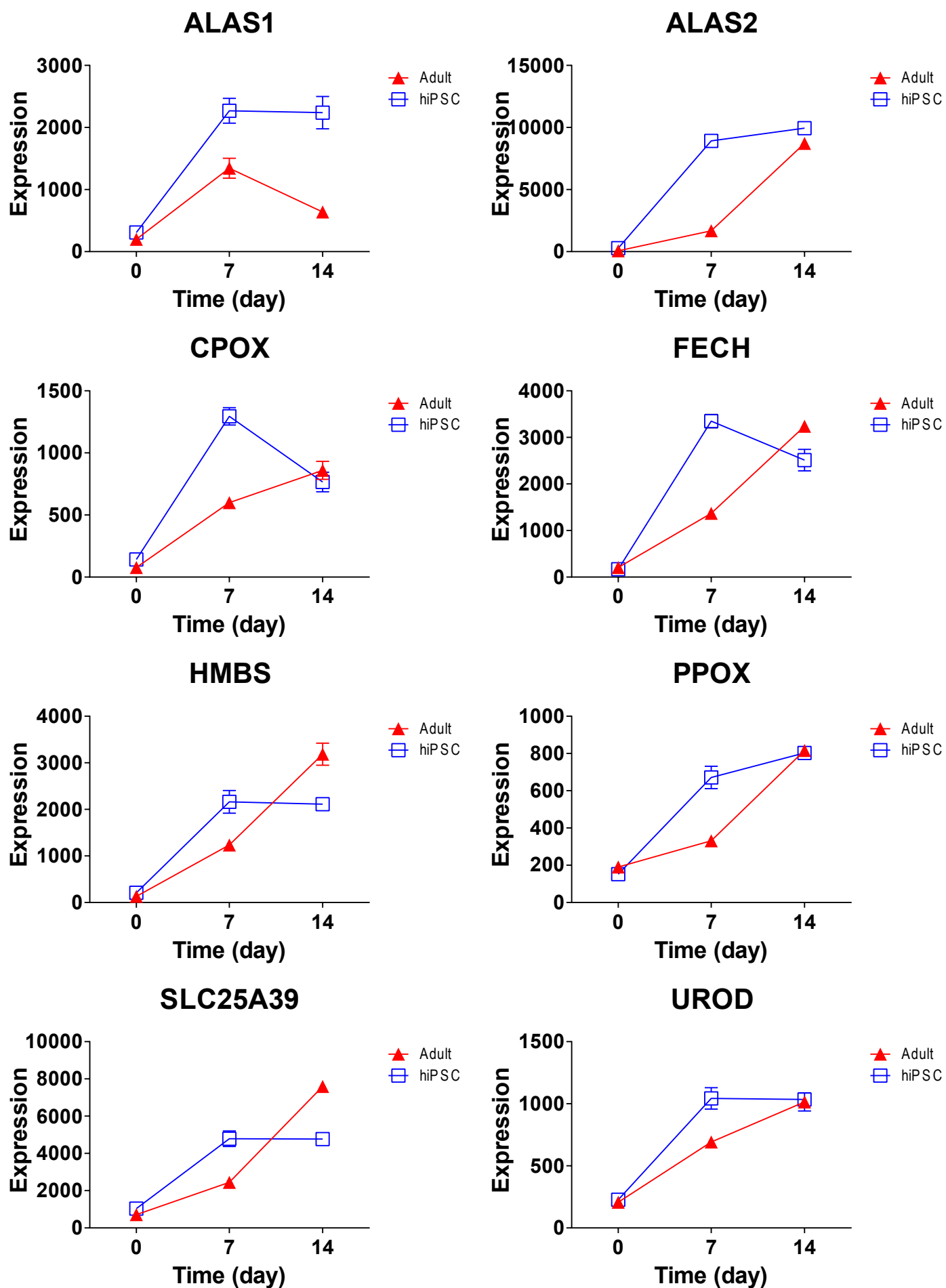

FIGURE S8

Supplement: Additional file 11: Figure S8. — Gene expression profiles for selected genes with roles in heme biosynthesis, in AB-erythroblasts and hiPSC-erythroblasts cultured in SEM-i. Mean expression +/- standard error of the mean is plotted. (PDF 295 kb) [file 12864_2016_3134_MOESM11_ESM.pdf]

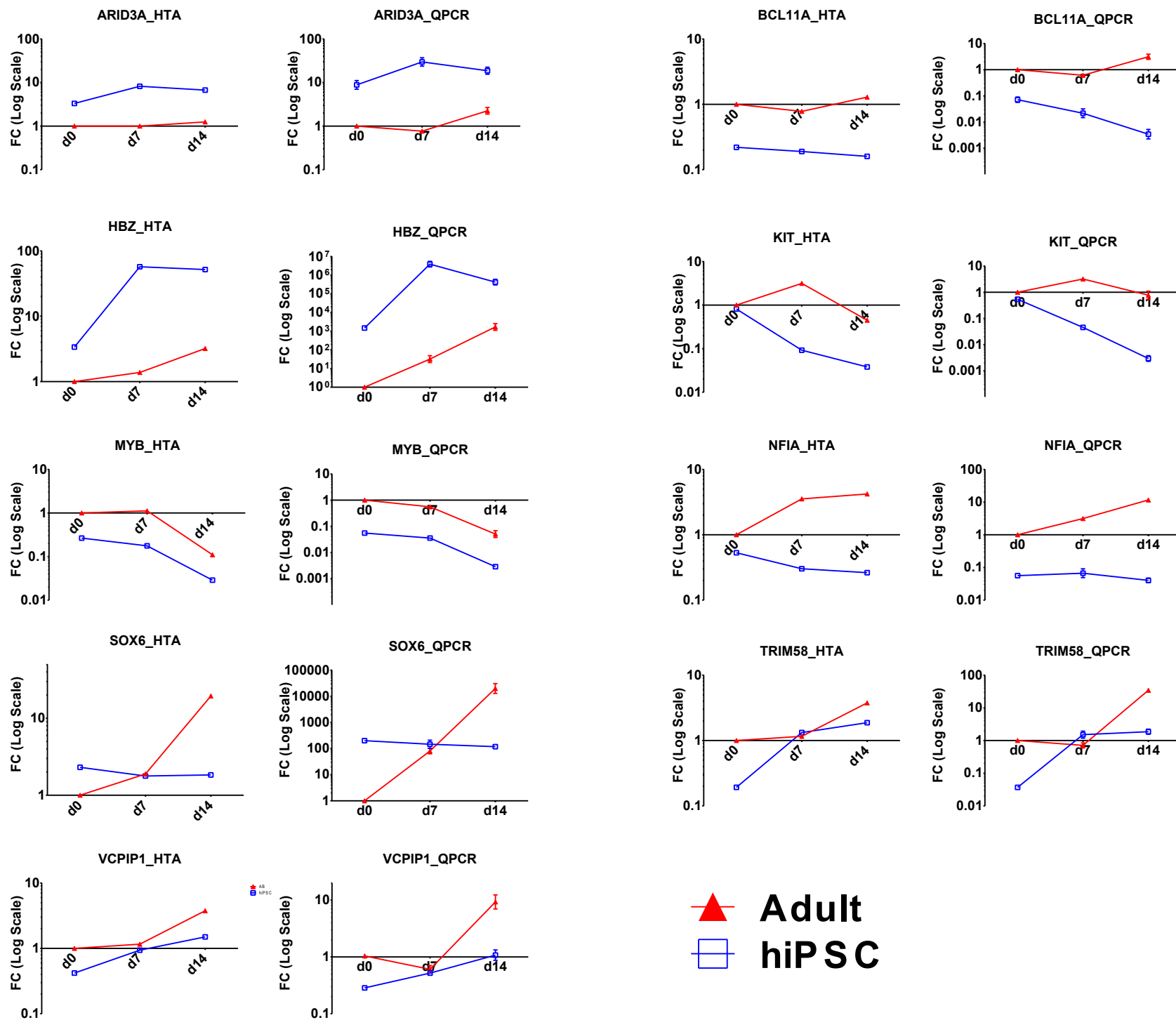

FIGURE S10B

Supplement: Additional file 13: Figure S10B. — Quantitative PCR validation of fold changes of selected genes observed by microarray. For each DE gene, the left hand graph shows fold changes (FC) relative to AB day 0 observed by microarray (“HTA”), and the right hand graph shows the same FC verified by QPCR (2 repeats of 2 representative samples in triplicate). A logarithmic scale (base 10) is used. Time is plotted on the x-axes: d0, day 0 (CD34+); d7, day 7 (SEM-i, CD71+); d14, day 14 (SEM-i, CD235a+). (PDF 383 kb) [file 12864_2016_3134_MOESM13_ESM.pdf]

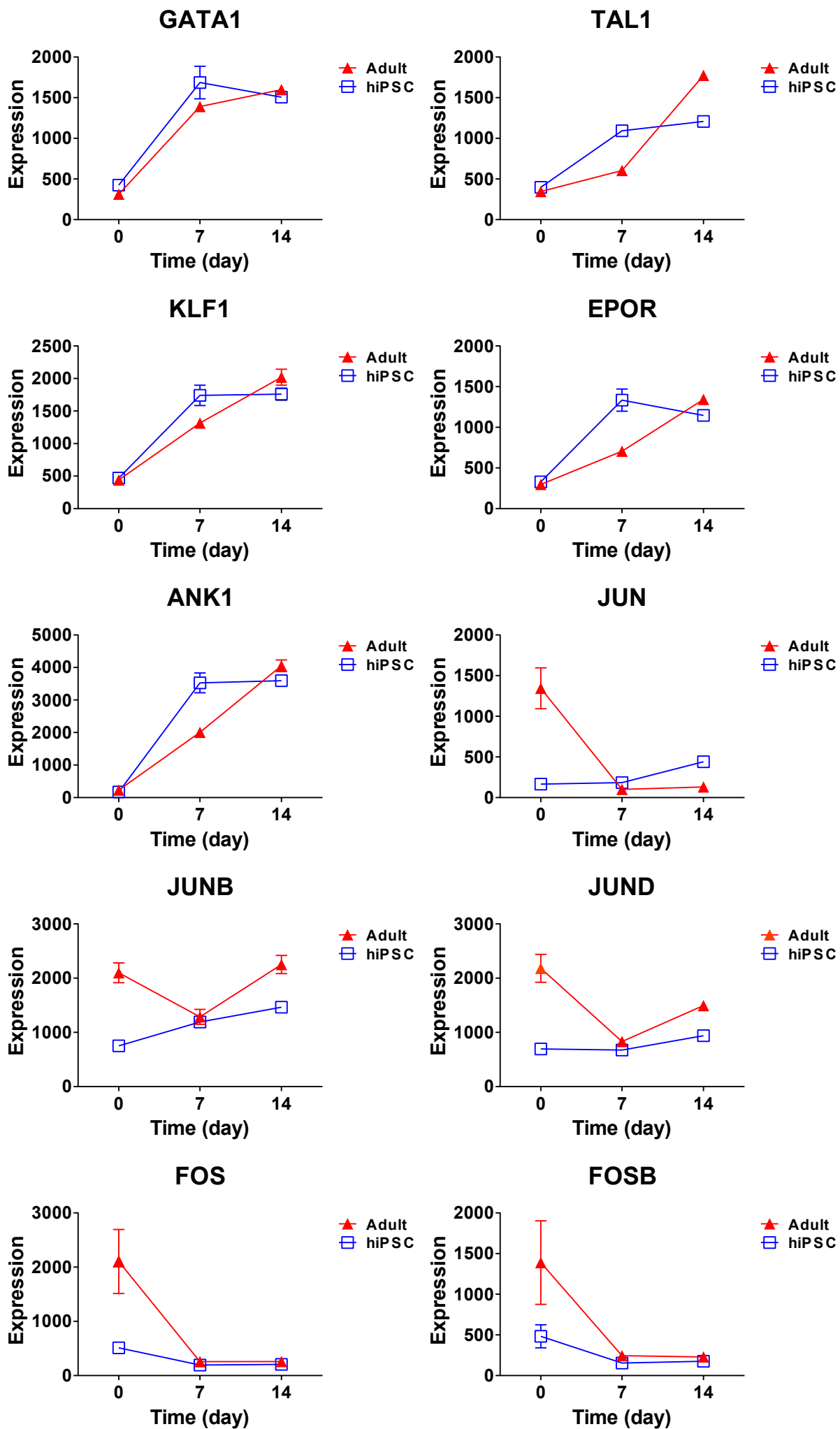

FIGURE S10A

Supplement: Additional file 14: Figure S10A. — Gene expression profiles for selected genes with roles in the control of erythropoiesis, in AB-erythroblasts and hiPSC-erythroblasts cultured in SEM-i. Mean expression ± standard error of the mean is plotted. (PDF 298 kb) [file 12864_2016_3134_MOESM14_ESM.pdf]

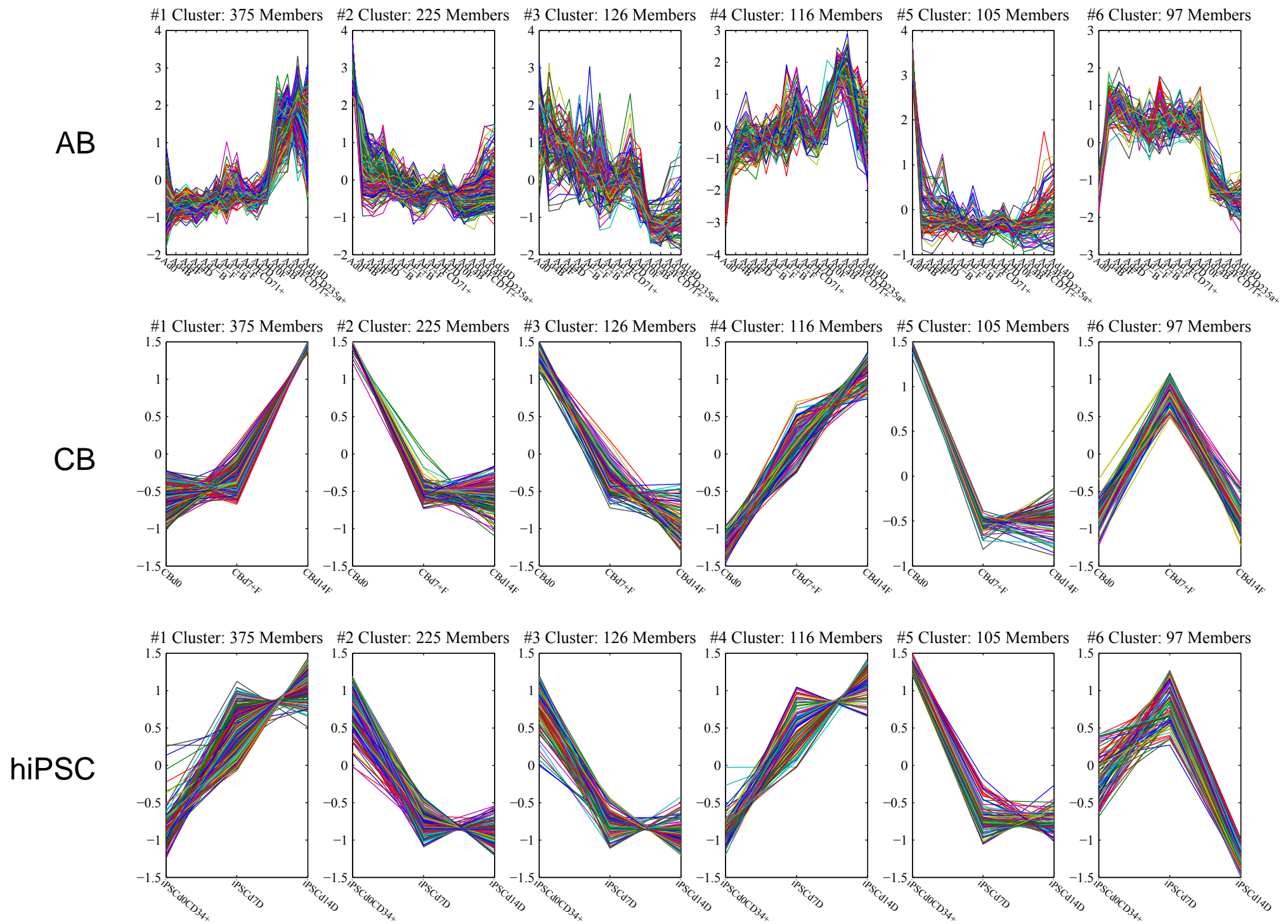

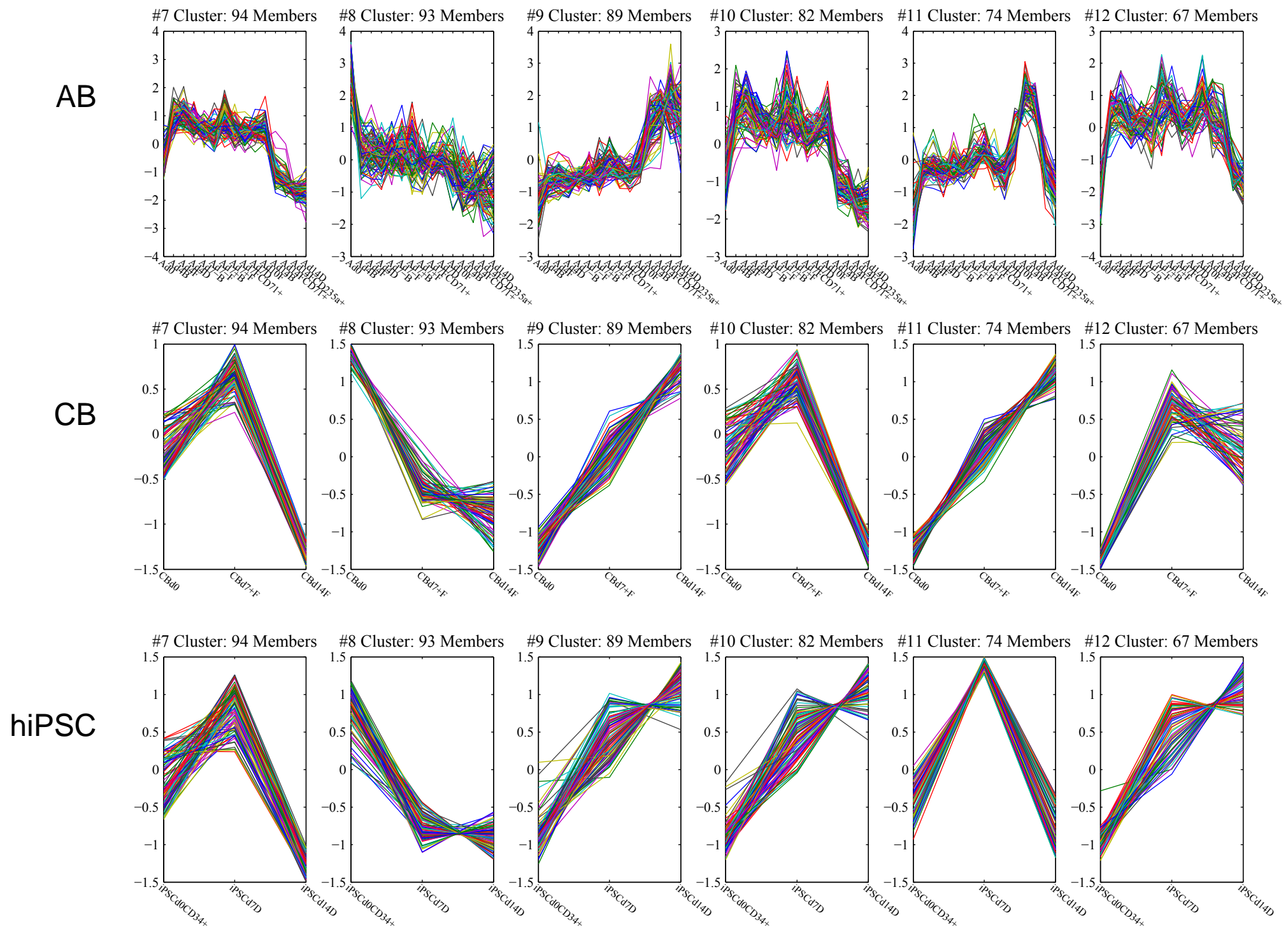

Figure S11

AB

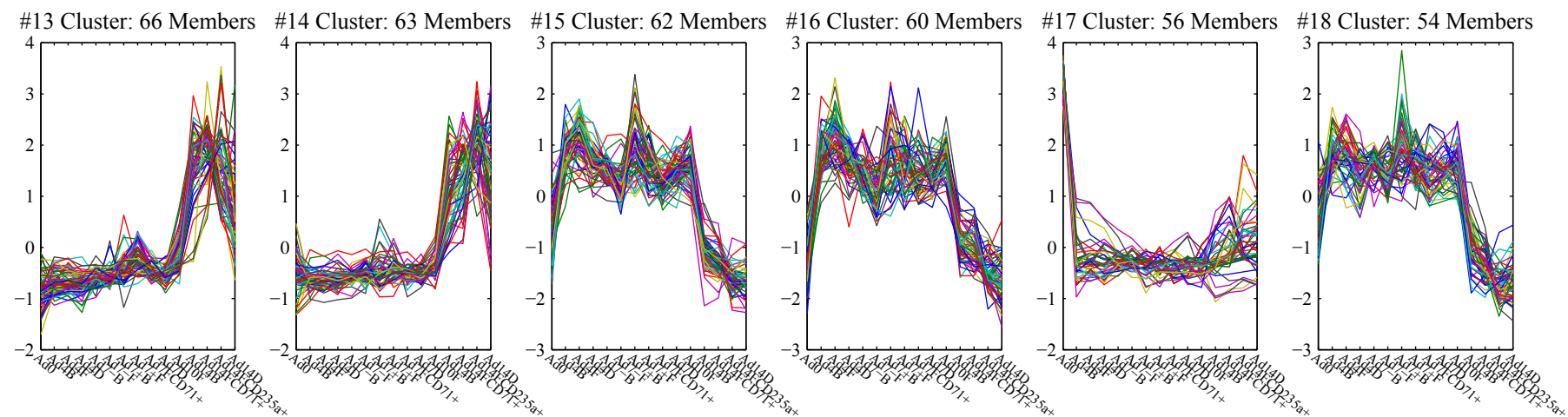

CB

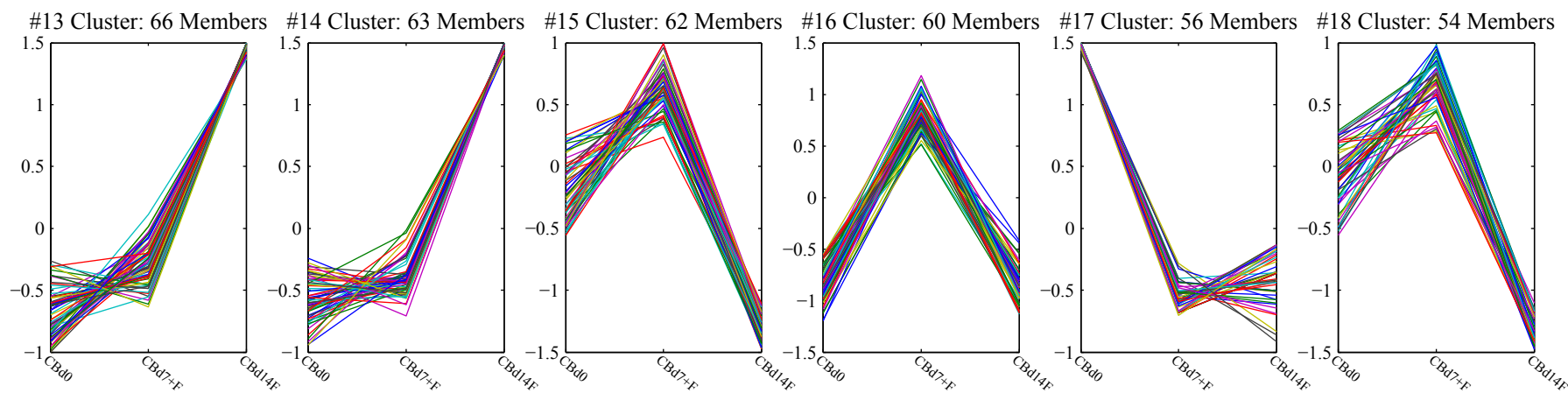

# hiPSC

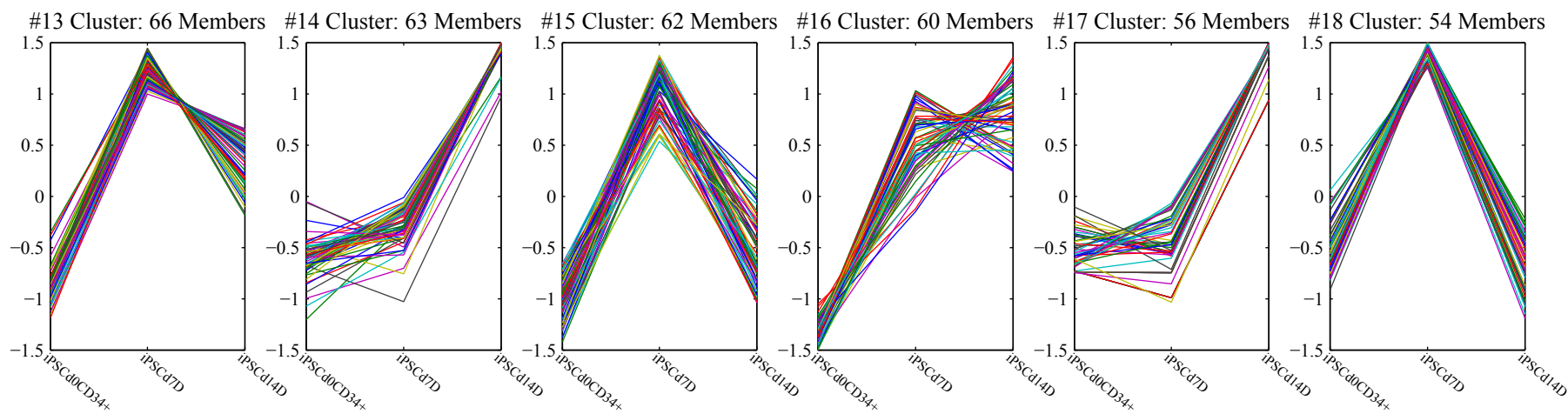

# Figure S11

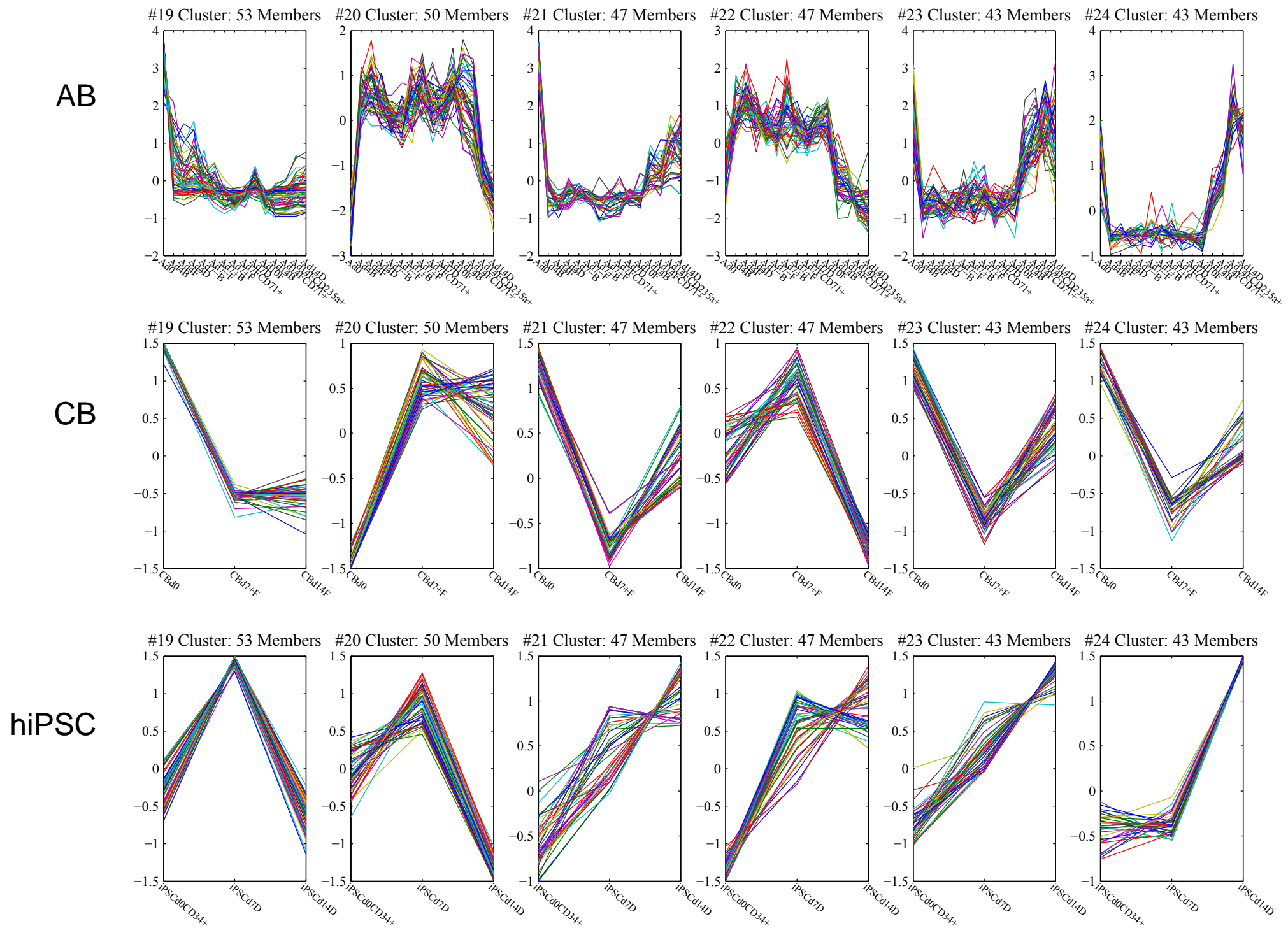

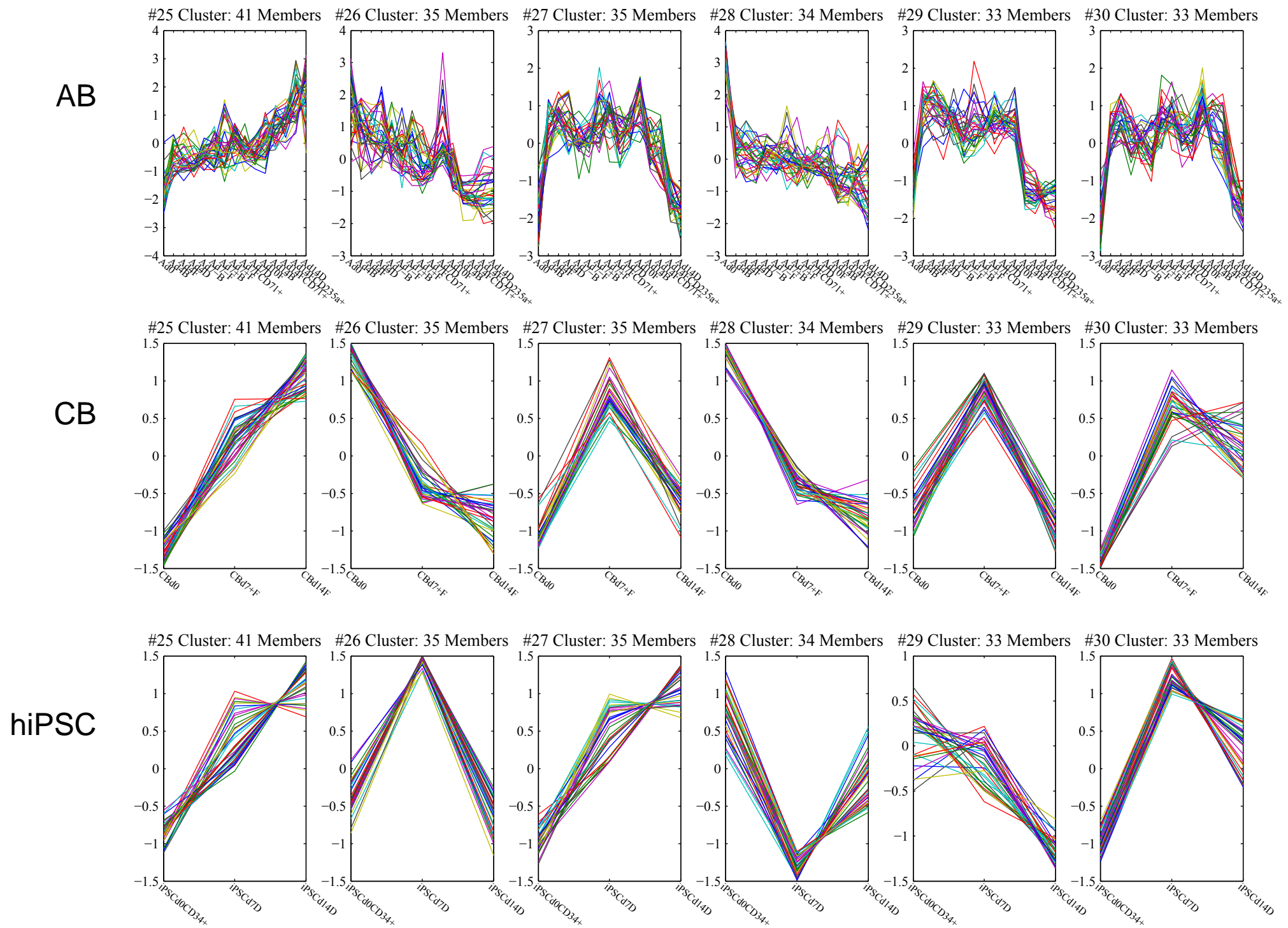

## Figure S11

Supplement: Additional file 15: Figure S11. — Expression profiles of genes within all clusters of >30 members derived from the SMART analysis of the global dataset are shown. Top row AB-erythroblasts, middle row CB-erythroblasts, bottom row hiPSC-erythroblasts. (PDF 287 kb) [file 12864_2016_3134_MOESM15_ESM.pdf]

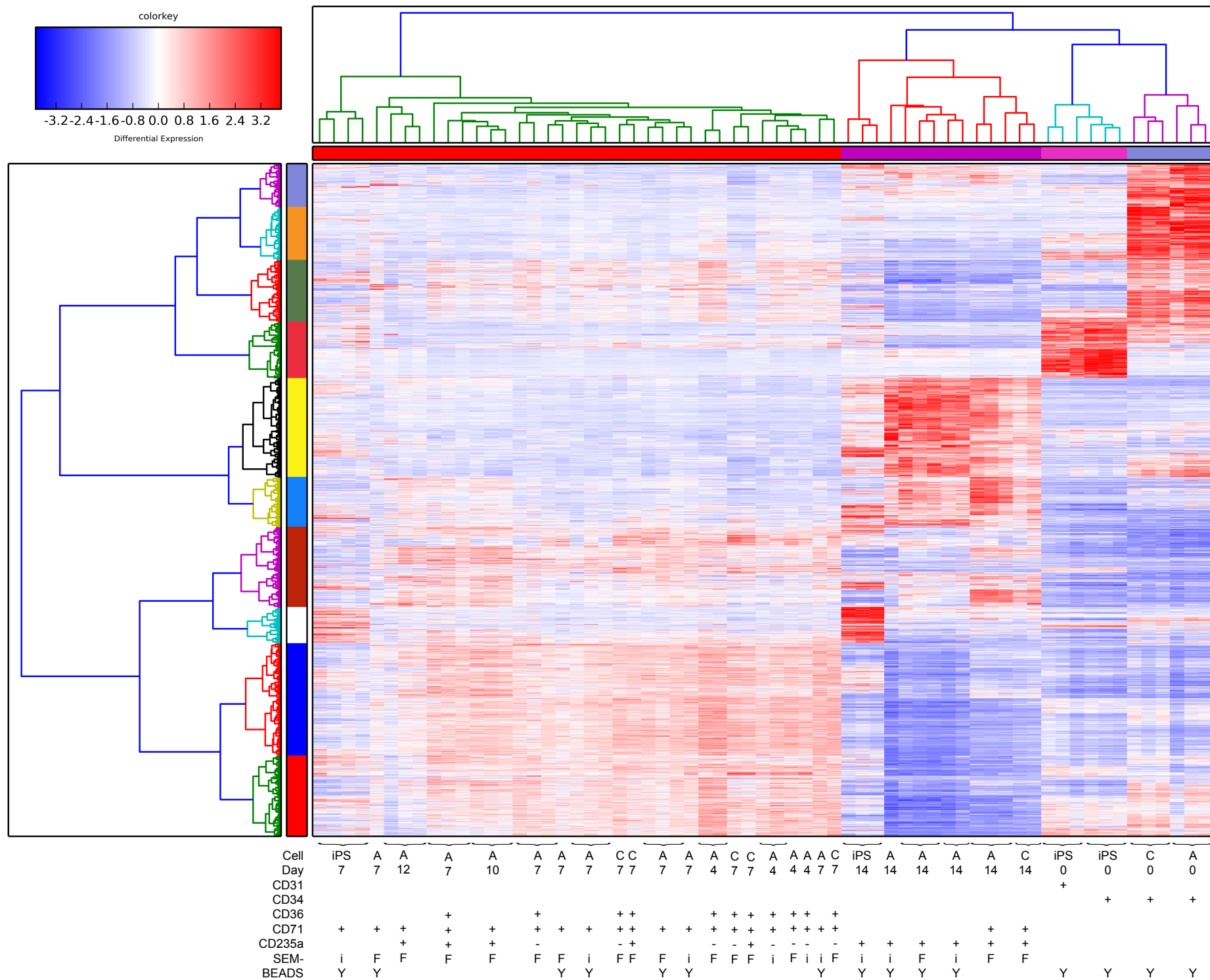

FIGURE S13

Supplement: Additional file 16: Figure S13. — HCL analysis of the union of DE genes from all cells of origin, in all media as described in the manuscript. HCL was prepared by Euclidean distance clustering by gene and by sample. A denotes adult, C denotes cord blood, and iPS denotes hiPSC-derived erythroid cultures. Samples isolated using paramagnetic beads rather than flow sorting are labelled. The colour bar on the left hand side denotes clusters of co-regulated genes. (PDF 5324 kb) [file 12864_2016_3134_MOESM16_ESM.pdf]

A Day 0

(i)

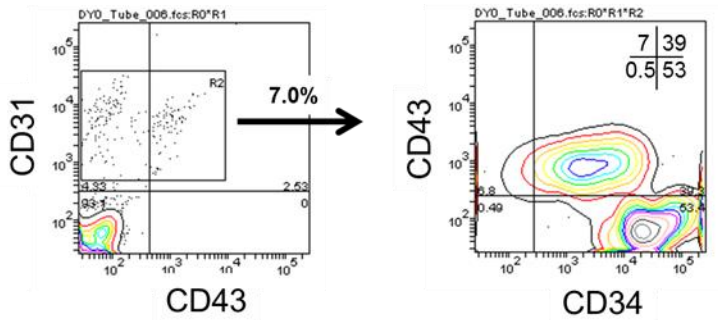

(ii)

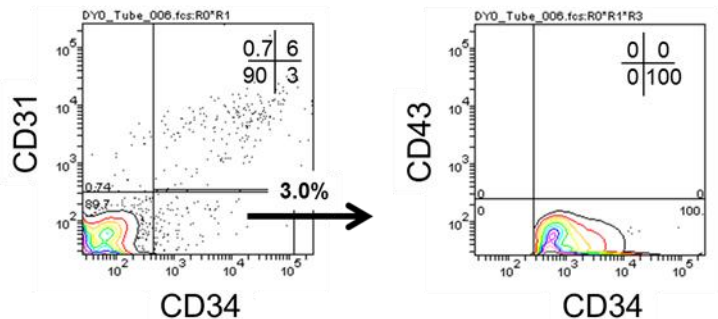

B Day 3

(i)

LIVE  
GATE

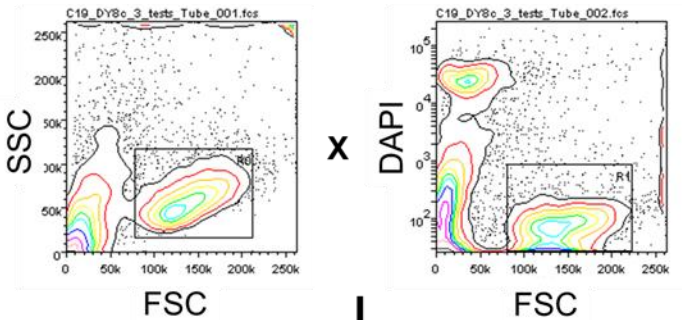

(ii)

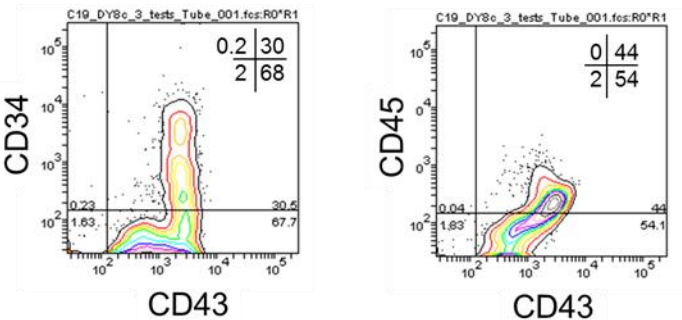

FIGURE S1

Supplement: Additional file 17: Figure S1. — The cell surface phenotype of progenitors derived from hiPSC co-culture on OP9 stromal cells. The proportion of cells that express cell surface proteins used to identify hematopoietic progenitors are shown in (A) on day 0 before transfer to SEM-i culture where (i) the CD31+ gate comprises more than 90 % CD34+ when CD43+ and CD43- are combined and (ii) only 3 % of CD34 + CD31- cells express the hematopoietic lineage marker CD43. On day 3 shown in (B) a Percoll gradient has removed OP9 cells so that (i) the viable DAPI negative culture grown on is (ii) enriched with approximately 98 % CD43+ hematopoietic cells and 30 % of cells are typically CD34+ some of which also express CD45. This indicates enrichment of hematopoietic multipotent progenitors during the first phase of culture in SEM-i as described previously by Dias et al. [18]. (PDF 144 kb) [file 12864_2016_3134_MOESM17_ESM.pdf]

## DAY 21

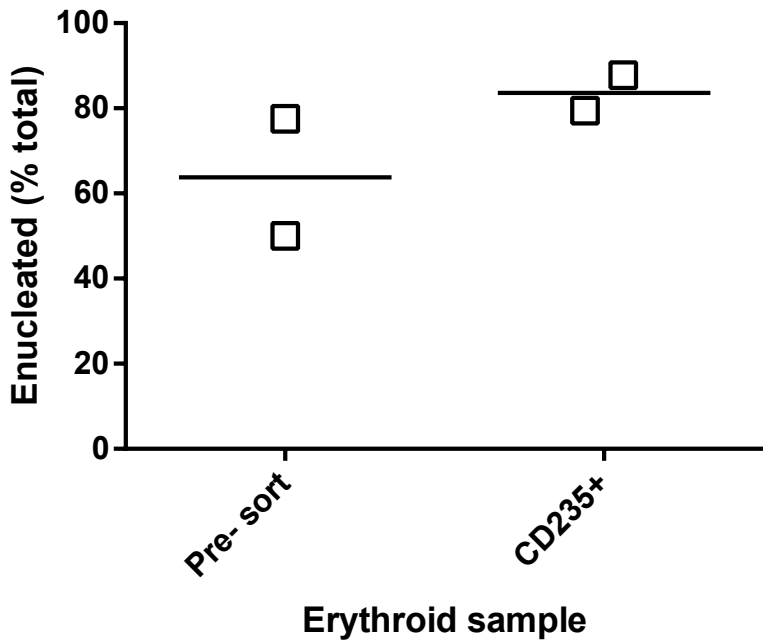

FIGURE S12

Supplement: Additional file 21: Figure S12. — Enucleated cells were enumerated from stained cytospin preparations prepared after extended culture period of 21 days in SEM-F. Samples were analysed before and after CD235a selection on paramagnetic beads. Observations from 2 independent cultures in SEM-i are shown from a minimum of 90 cells per culture condition. (PDF 21 kb) [file 12864_2016_3134_MOESM21_ESM.pdf]
