# Supplementary material for: Distinct gene expression program dynamics during erythropoiesis from human induced pluripotent stem cells compared with adult and cord blood progenitors
Source: BMC Genomics. 2016 Oct 21;17:817. doi: 10.1186/s12864-016-3134-z (PMC5073849; doi:10.1186/s12864-016-3134-z)
Supplement: Additional file 12: Figure S9. — A) Gene ontology analysis of genes regulated during the first 7 days of culture of AB-erythroblasts and hiPSC-erythroblasts in SEM-i. Significantly-scoring ontologies are shown, together with the more stringent corrected hypergeometric p-values produced by GeneCoDis, and the number of DE genes in each category from both cell types. B) Numbers of genes with roles in the cell cycle which are up-regulated in AB-erythroblasts and hiPSC-erythroblasts in SEM-i between days 0 and 7 are indicated, showing the numbers up-regulated from both sources or uniquely in each. (PDF 408 kb) [file 12864_2016_3134_MOESM12_ESM.pdf]

A

Cell of origin	Comparison	GO category	# of genes in category	Corrected hyp. p-value
Adult	Upregulated d0 to d7	Cell cycle (GO:0007049)	**96**	**3E-44**
hiPSC	Upregulated d0 to d7	Cell cycle (GO:0007049)	**29**	**9E-6**

B

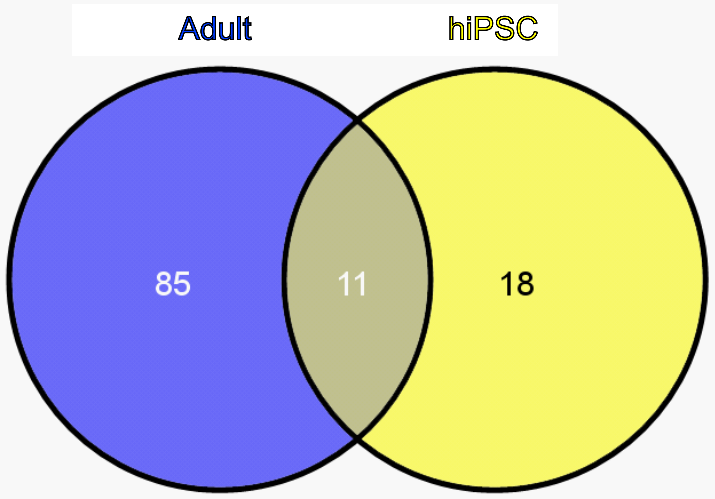

FIGURE S9
