# Supplementary material for: Distinct gene expression program dynamics during erythropoiesis from human induced pluripotent stem cells compared with adult and cord blood progenitors
Source: BMC Genomics. 2016 Oct 21;17:817. doi: 10.1186/s12864-016-3134-z (PMC5073849; doi:10.1186/s12864-016-3134-z)
Supplement: Additional file 18: Figure S3. — Isolation of erythroid precursors at defined stages of culture and derived from different sources of hematopoietic progenitors. A) Eryhthroblasts derived from cord blood CD34+ cells and cultured in SEM-F were enriched by FACs and gene expression compared to that from adult derived erythroblasts isolated using the same sort gates shown in Figure S2. The CD markers expressed by erythroblasts in the collected fractions are highlighted in blue. B) Erythroblast numbers derived from hIPSC progenitors grown in SEM-i were not sufficient for FACSorting and so populations based on time and phenotype were isolated using magnetic bead isolation. Day 7 erythroblasts were isolated using CD71 specific beads to target early precursors and Day 14 erythroblasts were isolated using beads specific to CD235a to target more mature erythroid cells. C) For comparison with hiPSC, CD34+ cells from Adult peripheral blood were cultured in the same media (SEM-i) and magnetic beads specific to CD71+ and CD235a + also used to isolate the populations shown on days 7 and 14 respectively. An example of sort purity achieved after D) use of FACS to obtain CD235a + CD71+ erythroblasts after 14 days of Adult HSC culture in SEM-F. The R5 sort gate was used to collect erythroblasts that were 94 % pure based on the expression of both CD235a and CD71. E) Magnetic beads conjugated to antibodies specific to CD71 were used on day 7 and beads specific to CD235a were used on day 14 of erythroid cultures in SEM-i. The purity of cells collected shown in R3 gate on day 7 or in R4 gate on day 14 is highlighted in blue. (PDF 190 kb) [file 12864_2016_3134_MOESM18_ESM.pdf]

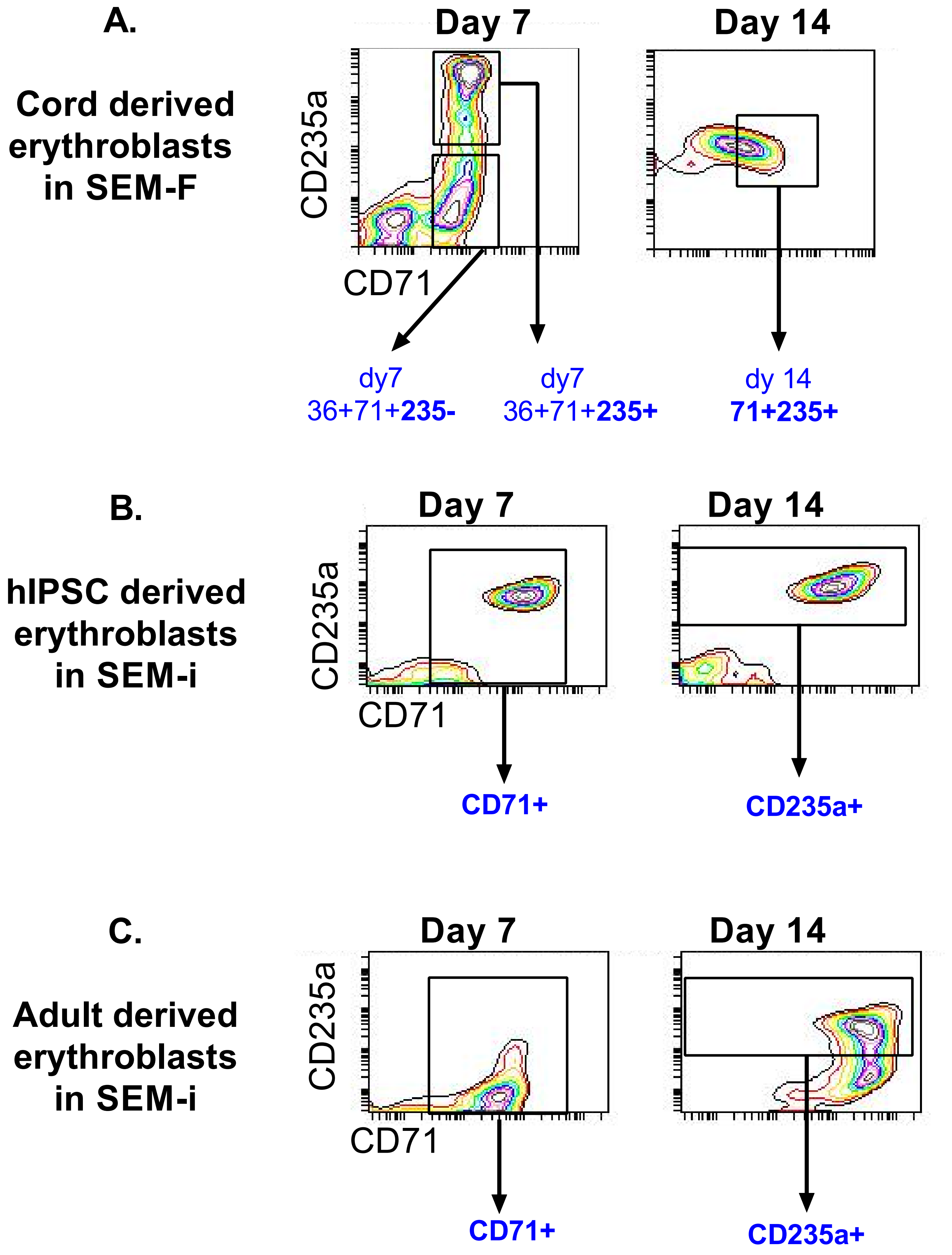
A.
Cord derived erythroblasts in SEM-F
Day 7
Day 14
CD235a
CD71
dy7
36+71+235-
dy7
36+71+235+
dy 14
71+235+
B.
hIPSC derived erythroblasts in SEM-i
Day 7
Day 14
CD235a
CD71
CD71+
CD235a+
C.
Adult derived erythroblasts in SEM-i
Day 7
Day 14
CD235a
CD71
CD71+
CD235a+

FIGURE S3

**D.**

**Sort Purity**

**Adult derived erythroblasts**

**Day 14**

**CD71+ CD235a+ (R5)**

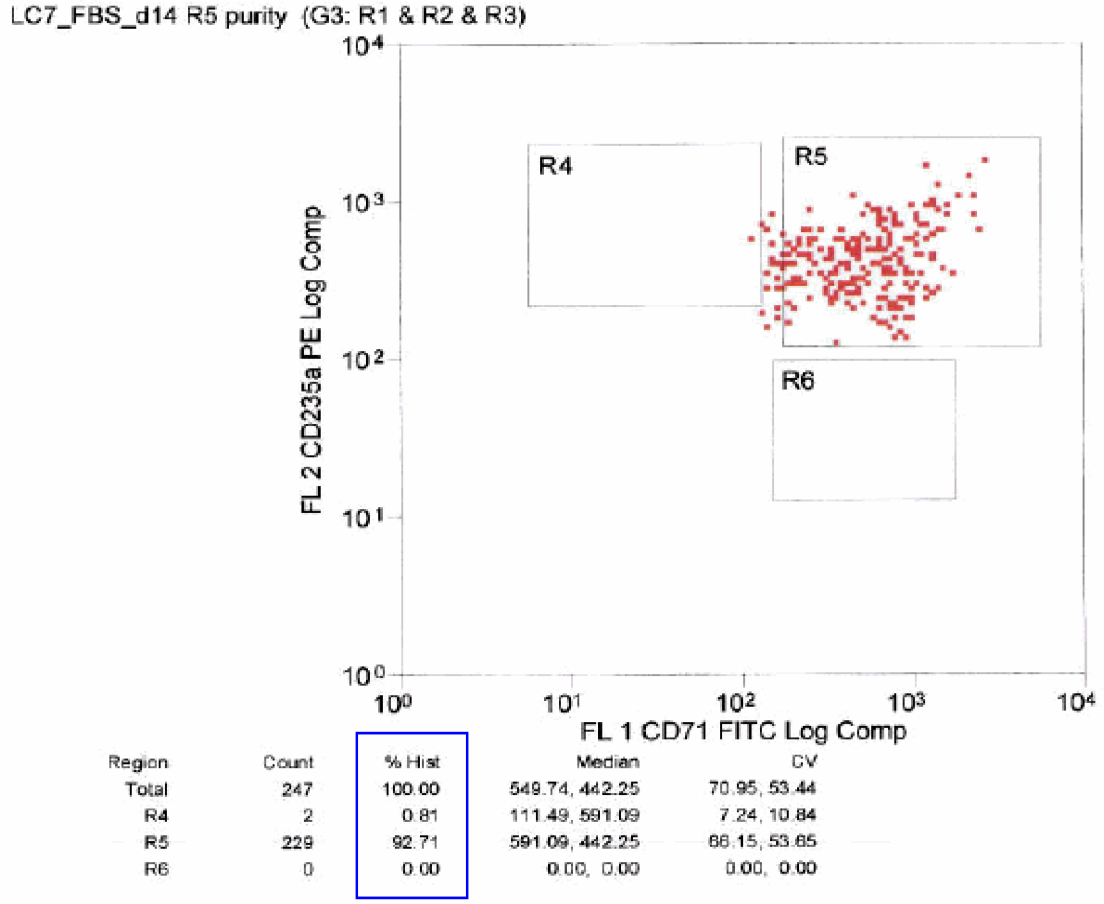


| Region | Count | % Hist | Median | CV |
|---|---|---|---|---|
| Total | 247 | 100.00 | 549.74, 442.25 | 70.95, 53.44 |
| R4 | 2 | 0.81 | 111.49, 591.09 | 7.24, 10.84 |
| R5 | 229 | 92.71 | 591.09, 442.25 | 66.15, 53.65 |
| R6 | 0 | 0.00 | 0.00, 0.00 | 0.00, 0.00 |

**E.**
**hIPSC derived Erythroblasts**

After magnetic column

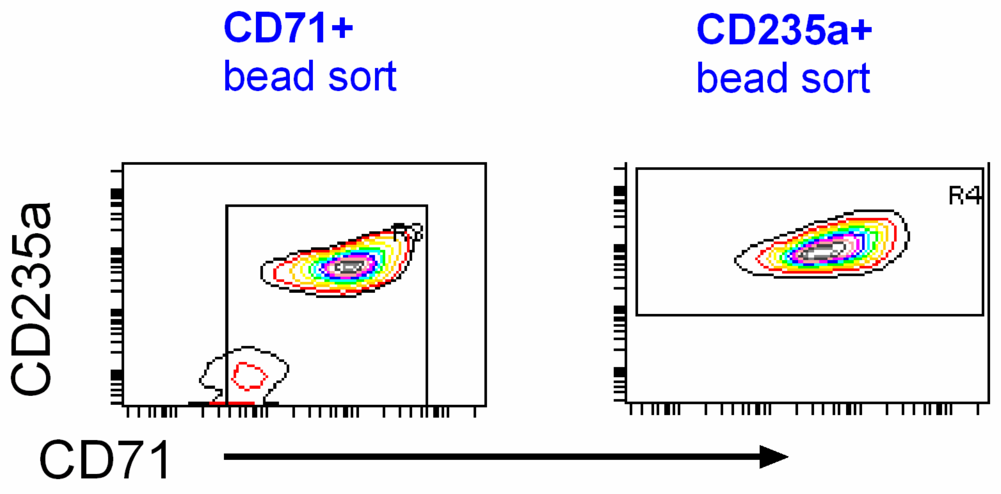


File: DY7_Tube_015.fcs
Gate: R0ANDR1
.... Region Stats ....
Region: %Gated
0: R0 100
1: R1 100
2: R2 85.83
3: R3 95.94
4: R4 86.29

File: DY14_Tube_010.fcs
Gate: R0ANDR1
.... Region Stats ....
Region: %Gated
0: R0 100
1: R1 100
2: R2 94.83
3: R3 96.35
4: R4 96.46

**FIGURE S3**
