# Supplementary material for: Distinct gene expression program dynamics during erythropoiesis from human induced pluripotent stem cells compared with adult and cord blood progenitors
Source: BMC Genomics. 2016 Oct 21;17:817. doi: 10.1186/s12864-016-3134-z (PMC5073849; doi:10.1186/s12864-016-3134-z)
Supplement: Additional file 19: — Tables S6, S7 and S8. The Euclidean distances from the PCAs shown in Figs. 1, 2 and 4 of manuscript. (PDF 96 kb) [file 12864_2016_3134_MOESM19_ESM.pdf]

Table S6 relating to Figure1A

	Ad0	Ad4	Ad7-	Ad7+	Ad10	Ad12	Ad14
Ad0	2.2453	41.7074	36.7059	39.7615	41.9005	38.6437	34.5571
Ad4	41.7074	2.4357	7.2265	13.9263	16.3334	16.2375	20.9985
Ad7-	36.7059	7.2265	2.3981	5.3486	7.6879	13.1318	35.9829
Ad7+	39.7615	13.9263	5.3486	2.448	2.8895	9.4553	32.9048
Ad10	41.9005	16.3334	7.6879	2.8895	2.4083	13.3944	56.9273
Ad12	38.6437	16.2375	13.1318	9.4553	13.3944	2.4299	29.2379
Ad14	34.5571	20.9985	35.9829	32.9048	56.9273	29.2379	2.4309

Table S7 relating to Figure2A

	Ad0	Ad7-	Ad7+	Ad14	CBd0	CBd7-	CBd7+	CBd14
Ad0	2.2055	18.9766	21.5794	16.6371	3.7572	21.8834	21.5561	16.0874
Ad7-	18.9766	2.4043	5.9359	48.3886	29.4614	5.0905	6.0852	38.0669
Ad7+	21.5794	5.9359	2.3699	34.306	35.1144	7.8387	5.514	26.5293
Ad14	16.6371	48.3886	34.306	2.4227	86.7417	128.4804	136.1507	25.5708
CBd0	3.7572	29.4614	35.1144	86.7417	2.434	45.739	46.1197	34.7566
CBd7-	21.8834	5.0905	7.8387	128.4804	45.739	2.4467	4.6173	18.3066
CBd7+	21.5561	6.0852	5.514	136.1507	46.1197	4.6173	2.4495	15.7422
CBd14	16.0874	38.0669	26.5293	25.5708	34.7566	18.3066	15.7422	2.4495

Table S8 relating to Figure 4A

	Ad0i	Ad7i BEADS	Ad14i BEADs	id0	id7i	id14i
Ad0i	2.151321	23.14539085	21.61662491	12.4059	16.27742	16.20017
Ad7i BEADS	23.14539	2.275996598	25.60912643	11.844	20.32124	27.12068
Ad14i BEADS	21.61662	25.60912643	2.4260222	15.75292	26.78691	29.62618
id0i	12.4059	11.84399784	15.75292059	2.331392	36.27215	44.01853
id7i	16.27742	20.32124431	26.78690601	36.27215	2.366381	6.694002
id14i	16.20017	27.12068119	29.62617741	44.01853	6.694002	2.397814
